# OMICS Profiling Identifies Signatures of Senescence in Osteogenesis Imperfecta Osteoblasts Counteracted by 4‐PBA

**DOI:** 10.1111/jcmm.71120

**Published:** 2026-04-06

**Authors:** Roberta Besio, Elisa Maffioli, Erika Palladino, Alessandra Sala, Nadia Garibaldi, Valerio Izzi, Antonella Forlino, Gabriella Tedeschi

**Affiliations:** ^1^ Department of Molecular Medicine, Biochemistry Unit University of Pavia Pavia Italy; ^2^ Department of Veterinary Medicine and Animal Sciences (DIVAS) University of Milan Lodi Italy; ^3^ Scuola Universitaria Superiore IUSS Pavia Pavia Italy; ^4^ Oulu Center for Cell‐Matrix Research and Biocenter Oulu University of Oulu Oulu Finland

**Keywords:** chemical chaperone, collagen, mass spectroscopy, osteogenesis imperfecta, secretome, senescence

## Abstract

Mutations in collagen I are the most common cause of osteogenesis imperfecta (OI), leading to delayed protein folding and structurally abnormal molecules. While some aberrant collagen is secreted into the extracellular matrix (ECM), impairing bone quality, a significant fraction is retained intracellularly, disrupting osteoblast homeostasis. 4‐phenylbutyrate (4‐PBA) has been shown to improve osteoblast function and ECM composition in OI models. To investigate the intracellular consequences of mutant collagen retention and the mechanisms of 4‐PBA, we analysed the secretome and transcriptome of two dominant OI mouse models, *Col1a1* ^*+/G349C*^ and *Col1a2* ^*+/G610C*^. MS/MS proteomic analysis of conditioned media revealed senescence‐associated secretory phenotype proteins, together with components linked to altered cytoskeletal organization and cell adhesion. Transcriptomic analysis identified *P53* as a central hub gene, supporting premature senescence activation. Increased senescence‐associated β‐galactosidase activity, elevated expression of the cyclin‐dependent kinase inhibitor *P16*, and reduced *Ki67* levels further supported a senescent phenotype. Notably, senescence‐associated proteins were absent from the secretome following 4‐PBA treatment, which also modulated cytoskeletal and adhesion‐related protein expression. Moreover, 4‐PBA significantly reduced senescence marker expression and decreased the number of senescent cells. Overall, these findings indicate that cellular senescence underlies osteoblast dysfunction in OI and uncover a novel contribution of 4‐PBA to osteoblast homeostasis.

**Abbreviations**
4‐PBA: 4‐phenylbutyrate
APP: amyloid‐precursor protein
BSA: bovine serum albumin
CAN: acetonitrile
CGP: chemical and genetic perturbations
CRYAB: crystallin alpha B
DA: differential abundance
ECM: extracellular matrix
ER: endoplasmic reticulum
FBS: fetal bovine serum
Foxo3: forkhead box O3
GOBP: gene ontology biological process
GOMF: gene ontology molecular function
H: hallmark
LC‐ESI‐MS/MS: liquid chromatography–electrospray ionization–tandem mass spectrometry
LFQ: label free quantification
Lmnb1: laminin b1
MSigDB: molecular signatures database
OB: osteoblast
OI: osteogenesis imperfecta
PTM: post translational modification
SASP: senescence‐associated secretory phenotype
SA‐β‐Gal: senescence‐associated beta‐galactosidase
UPR: unfolded protein response
α‐MEM: α‐modified Eagle's medium

## 1 Introduction

Osteogenesis imperfecta (OI) is an inherited skeletal disorder mainly caused by autosomal dominant mutations in the genes encoding type I collagen. The disease is most commonly associated with glycine substitutions in the collagen I α chains, which delay triple‐helix folding, leading to increased post‐translational modifications and an abnormal collagen structure [1, 2, 3]. Mutant collagen is partially secreted into the extracellular matrix (ECM) where it impairs ECM assembly and quality, and partially retained in the endoplasmic reticulum (ER) perturbing osteoblast (OB) homeostasis [4, 5, 6]. To cope with the resulting ER stress, OI cells activate the adaptive unfolded protein response (UPR), a dynamic signalling network that orchestrates the recovery of homeostasis or triggers apoptosis, depending on the level of damage [7]. Thus, the response to ER stress becomes an important modifier of OI severity. Of note, chemical chaperones had been proposed in clinical trials to treat diseases caused by intracellular accumulation of misfolded proteins [8]. Among them, 4‐phenylbutyric acid (4‐PBA), approved by the EMA and FDA for urea cycle disorders, is appealing as a repurposing drug to treat collagen‐related disorders associated with ER stress [8, 9]. Our group proved that 4‐PBA is beneficial to restore OI OB homeostasis and to improve ECM quantity and quality [6, 10]. At the cellular level, 4‐PBA prevented intracellular accumulation of collagen and increased protein secretion, reducing aggregates within the mutant cells and normalizing ER morphology. At the extracellular level, the drug increased collagen incorporation into the matrix and promoted OB mineral deposition [6]. Recently, an in vivo study using the *Col1a2* ^*+/G610C*^ murine model of moderately severe OI revealed that 4‐PBA treatment enhanced longitudinal bone growth [11], while in the A*ga2* ^*+/−*^ mouse, a classical model of severe OI due to a *Col1a1* structural mutation, the drug decreased fracture incidence and increased body length and weight, femoral bone volume and cortical thickness [11, 12]. Another study using an *Atg7* knock‐out mouse model with an OB‐specific mutation impairing autophagy, causing ER stress and compromised bone formation and mineralization, confirmed the 4‐PBA beneficial effect on bone forming cells' metabolism [13]. Of relevance, it has been reported that 4‐PBA targets specifically the COPII coat protein P24, a component of the secretory pathway necessary for the translocation of large proteins from the ER to the Golgi, promoting COPII packaging of ER proteins, stimulating their sorting, reducing trafficking stringency and thus promoting secretion [14]. Furthermore, our group also demonstrated that by doing that, 4‐PBA is also improving general cell secretion [15]. The secretome, the set of molecules secreted into the extracellular space, including both soluble proteins and extracellular vesicles, is indeed cell specific and represents the cell fingerprint, with extracellular vesicles content reflecting the intracellular organization and metabolism of the parent cell [16, 17, 18, 19]. It plays a crucial role in mediating cell‐to‐cell communication both locally and systemically to maintain the cellular physiological homeostasis while fluctuations in its composition are reported in pathological conditions [20, 21]. Indeed, the cross talk among osteoblasts, osteocytes, and osteoclasts, necessary to guarantee skeletal homeostasis, is strictly coordinated by bone cell‐derived secretome [22, 23].

Given its relevance in determining bone formation and maintenance, here we applied proteomic analyses to investigate the OBs secretome impact on OI pathology, as well as to clarify the effect of 4‐PBA in primary OI cells. Transcriptomic analyses of the ER‐stress–activated cellular response, together with cellular assays, were used to complement the secretome data by elucidating the intracellular molecular changes. To analyse the effect of mutations on both collagen I α chains, primary OBs were isolated from the OI *Col1a1* ^*+/G349C*^ (Brtl) and the *Col1a2* ^*+/G610C*^ (Amish) murine models, carrying a glycine substitution in *Col1a1* and *Col1a2* genes respectively. Both mice reproduce growth deficiency, lower bone mass, and altered bone properties, which are typical of individuals affected by OI [6], thus representing unique translational models for dissecting the molecular basis of OI pathology and to clarify the mechanism underlying the effect of new therapeutic strategies.

## 2 Experimental Procedures

### 2.1 Experimental Design and Statistical Rationale

To analyse the secretome and transcriptome changes induced by collagen I mutations and by incubation with the chemical chaperone 4‐PBA, three biological replicates were collected from primary osteoblasts isolated from two OI murine models, the *Col1a1* ^*+/G349C*^ and the *Col1a2* ^*+/G610C*^ mice. This setup results in eight conditions (*Col1a1* ^*+/+*^, *Col1a1* ^*+/+*^ 4‐PBA, *Col1a1* ^*+/G349C*^, *Col1a1* ^*+/G349C*^ 4‐PBA, *Col1a2* ^*+/+*^, *Col1a2* ^*+/+*^ 4‐PBA, *Col1a2* ^*+/G610C*^, *Col1a2* ^*+/G610C*^ 4‐PBA), comprising 24 samples. The protein expression profile and quantitation were evaluated by LC‐ESI‐MS/MS before and following 4‐PBA incubation, while gene expression was analysed by array and qPCR. OBs isolated from wild type (WT) littermates were designated as the control condition. Sex was not considered as a biological variable. The statistical analysis is described in the statistical analysis section.

### 2.2 Animals

CD1/129Sv/B6 *Col1a1* ^+/G349C^ mice, carrying a α1(I)‐G349C substitution [24], their WT littermates *Col1a1* ^+/+^ and CD1/CH3/B6 *Col1a2* ^*+/G610C*^ mice, carrying a α2(I)‐G610C substitution, and their WT littermates *Col1a2* ^*+/+*^ were used [25]. The mice were maintained under standard experimental animal care in the ‘Centro interdipartimentale di servizio per la gestione unificata delle attività di stabulazione e di radiobiologia’—Istituto Golgi‐Spallanzani of the University of Pavia (Italy). All the experiments were approved by the Office for the Animals Welfare (OPBA) of the University of Pavia and by the Italian Ministry of Health (Protocol N 8/2019‐PR), complied with the ARRIVE guidelines and were carried out in accordance with the EU Directive 2010/63/EU for animal experiments. Mice genotyping was performed by PCR on DNA isolated from tail biopsies as previously reported [6].

### 2.3 Calvarial Osteoblast Culture and 4‐PBA Incubation

Murine OBs were isolated from 2 to 4 days old WT and mutant pups as previously reported [6]. Cells derived from a minimum of three animals of the same genotype were combined to obtain a sufficient cell yield for experimental procedures. Three independent culture replicates were prepared. Cells were cultured in α‐Modified Eagle's Medium (α‐MEM, Lonza) supplemented with 10% fetal bovine serum (FBS, Euroclone), 50 μg/mL sodium ascorbate (Fluka), 4 mM glutamine (Euroclone), 100 μg/mL penicillin and streptomycin (Euroclone) at 37°C in a humidified atmosphere containing 5% CO_2_. Cells were used only at passage 1. Identical cell density (1.5 × 10^4^/cm^2^) was used for all the experiments. Cells were cultured with no media change with the addition every other day of 50 μg/mL ascorbic acid and harvested after 5 days, reproducing the previously used condition in which ER stress activation was detected in the mutant [6]. 5 mM 4‐PBA was added 16 h before the harvest. The dose was chosen based on previous in vitro studies showing a positive effect on OB homeostasis [6].

### 2.4 Collection of Conditioned Media for LC/ESI‐MS/MS Analysis

OBs were plated in two 10 cm petri dishes for each condition. After three washes in α‐MEM without FBS, the medium was changed to α‐MEM without FBS, 50 μg/mL sodium ascorbate, 4 mM glutamine, 100 μg/mL penicillin and streptomycin with and without 5 mM 4‐PBA, and cells were incubated for 16 h. Conditioned medium, containing both soluble proteins and extracellular vesicles, was harvested and centrifuged at 2000 g for 10 min at 4°C to remove cells, cellular debris and apoptotic bodies. Proteins were quantified by quantum protein bicinchoninic protein assay kit (Euroclone). Bovine serum albumin (BSA) (Sigma‐Aldrich) was used as standard. At least 1 mg of total protein was obtained for each sample. Samples were kept frozen until use.

### 2.5 Liquid Chromatography Electrospray Ionization Tandem Mass Spectrometry (LC/ESI‐MS/MS) Analysis

Proteins were precipitated with 10% trichloroacetic acid for 2 h on ice [26], reduced, carbamidomethylated, and digested with trypsin for 16 h at 37°C using a protein trypsin ratio of 20:1 [27]. Nano LC‐ESI‐MS/MS analysis was performed on a Dionex UltiMate 3000 HPLC System directly connected to an Orbitrap Fusion Tribrid mass spectrometer (Thermo Fisher Scientific) by a nanoelectrospray ion source. Peptide mixtures were enriched on a 75 μm ID × 150 mm EASY‐Spray PepMap RSLC C18 column (Thermo Fisher Scientific) and separated using the LC gradient: 1% acetonitrile (ACN) in 0.1% formic acid for 10 min, 1%–4% ACN in 0.1% formic acid for 6 min, 4%–30% ACN in 0.1% formic acid for 147 min, and 30%–50% ACN in 0.1% formic for 3 min at a flow rate of 0.3 μL/min. Orbitrap MS spectra were collected over an m/z range of 375–1500 at a resolution of 120,000, operating in a data‐dependent mode with a cycle time of 3 s between master scans. HCD MS/MS spectra were acquired in Orbitrap at a resolution of 15,000 using a normalized collision energy of 35%, and an isolation window of 1.6 m/z. Dynamic exclusion was set to 60 s. Rejection of +1 and unassigned charge states were enabled. Data acquisition was controlled by Xcalibur 2.0 and Tune 2.4 software [28]. The mass spectrometry proteomics data have been deposited to the ProteomeXchange Consortium via PRIDE (PXD059591) [29].

Mass spectra were analysed using MaxQuant software (version 1.6.10) [30]. The spectra were searched by the Andromeda search engine against the murine UniProt sequence database (released 22 September 2021). Protein identification required at least one unique or razor peptide per protein group. The initial maximum allowed mass deviation was set to 10 ppm for monoisotopic precursor ions and 0.5 Da for MS/MS peaks. Enzyme specificity was set to trypsin, defined as C‐terminal to Arg and Lys excluding Pro, and a maximum of two missed cleavages was allowed. Carbamidomethylcysteine was set as a fixed modification, while N‐terminal acetylation, Met oxidation and Asn/Gln deamidation were set as variable modifications. Quantification in MaxQuant was performed using the built in XIC‐based label free quantification (LFQ) algorithm using fast LFQ. The required false positive rate was set to 1% at the peptide and 1% at the protein level, and the minimum required peptide length was set to 7 amino acids [31, 32].

### 2.6 Protein Bioinformatic Analyses

#### 2.6.1 Protein Classification

Classification of the secreted proteins was performed with the following databases: Uniprot, using the key words ‘secreted’, ‘extracellular location’ (GOCC); ExoCarta and Vesiclepedia databases [33, 34]; DAVID 6.8, using the key words ‘extracellular region, extracellular exosome, secretory granule, vesicle etc.’ (GOCC term), ‘Signal, secreted, glycoprotein’. Fischer's exact test *p* ≤ 0.05 and at least 2 counts; PANTHER 16.0 using the key words ‘extracellular region, vesicle etc’ (GOCC term). Fischer's exact test (*p* ≤ 0.05) and at least 2 counts.

All the proteins identified as ‘non‐secreted’ were searched with the prediction programs Secretome P 2.0 and Signal P 6.0 [35].

In the analysis, proteins were assigned as “secreted” if they were identified by at least one bioinformatics software.

#### 2.6.2 Protein Functional Analyses

“Secreted” proteins were further analysed to understand the functional significance of variations in protein expression, underlying the regulation of entire cellular processes or biochemical pathways by CLUEGO 2.5.7 (Cytoskape) [36], Panther 16.0 [37], DAVID 6.8 [38], IPA (released April 2022) [39] and SASP Atlas database [40, 41].

In particular, the data of *Col1a1* ^+/G349C^ and *Col1a2* ^+/G610C^ samples were analysed separately to understand in each model the impact of the genotype and the effect of 4‐PBA. In each model the variation among the groups was analysed by one‐way ANOVA followed by post hoc tests with the Bonferroni's correction (*p*‐value ≤ 0.05). The proteins ANOVA significant were further analysed by Cytoskape, Panther, DAVID and IPA software to classify them based on gene ontology biological process (GOBP), gene ontology molecular function (GOMF) and pathway (at least 3 genes count) while the analysis by senescence‐associated secretory phenotype (SASP) Atlas database was performed to identify SASP‐related proteins.

### 2.7 Gene Expression Analysis

Total RNA was extracted from OBs plated in 24 well plate from 3 independent cell preparations per genotype in absence or presence of 4‐PBA with QIAzol Lysis Reagent (Qiagen). cDNA synthesis was performed using RT^2^ First Strand kit (Qiagen) according to the manufacturer's protocol. *P53*, *P16*, *Ki76*, *Lmnb1 and Foxo3* gene expression was analysed by RT‐qPCR. *Gapdh* was used as normalizer. Relative expression was calculated by the ΔΔCt method. All reactions were performed in triplicate. QuantStudio3 thermocycler (Thermofisher) using powerUp syber green master mix (Applied Biosystems) was used. Primers will be available upon request.

### 2.8 Gene Expression Normalization, Enrichment Analyses, and Network Construction

Bioinformatic analysis on transcriptomic data from UPR, autophagy and apoptosis arrays carried out in [6] was performed. Gene expression data were normalized independently for each sample to account for inter‐sample variability and ensure comparability across experimental conditions. To identify differentially expressed genes, pairwise comparisons between groups were performed using the non‐parametric Mann–Whitney U test. Functional enrichment analyses were conducted to contextualize the gene expression changes within broader biological processes. Specifically, Gene Ontology (GO) enrichment and pathway analyses were performed using: Profiler and the Molecular Signatures Database (MSigDB), including both the Hallmark (H) and Chemical and Genetic Perturbations (CGP) gene sets, using the FGSEA algorithm as previously described [42, 43]. For GO analyses, the top 100 enriched terms per condition were retained for downstream interpretation.

To infer molecular interaction patterns, condition‐specific networks were constructed by overlaying significantly upregulated genes onto a composite background interactome. This reference network integrated curated high‐confidence protein–protein interaction (PPI) datasets—including the Human Reference Interactome, NicheNet, and SIGNOR databases [44, 45, 46]—alongside supplementary low‐confidence sources such as STRINGdb and BioGRID [47, 48]. Direct interactors of the upregulated genes were extracted from this network to generate interaction maps reflective of condition‐specific signalling dynamics. Gene‐to‐protein identifier mapping, including cross‐species ortholog conversions where necessary, was performed using the BioMart platform [49].

### 2.9 Senescence‐Associated β‐Galactosidase Staining

Senescence‐associated beta‐galactosidase (SA‐β‐Gal) staining of primary osteoblasts was performed in 24 well plates according to the manufacturers' instructions (Cell Signalling Technology), with a cell confluence at staining around 60%. SA‐β‐Gal activity was identified based on positively stained blue cells. The number of senescent cells was normalized to DNA content, which was extracted from an adjacent well cultured under the same experimental conditions. Three biological replicates were analysed for each condition.

### 2.10 Statistical Analysis

Biological triplicates for each experiment were performed. Quantitative variables were expressed as mean ± SD. One‐way ANOVA was applied to evaluate genotype and treatment effect. A *p*‐value ≤ 0.05 was considered significant. For the proteomic data, statistical analyses were performed using the Perseus software (version 1.5.5.3). Only proteins present and quantified in 3 out of 3 biological repeats in at least one experimental condition were considered. Proteins not meeting this minimum valid‐value criterion were classified as exclusively expressed. No missing‐value imputation was applied, and only experimentally observed LFQ intensity values were used for Venn diagram–based classification and ANOVA.

## 3 Results

### 3.1 Secretome Protein Profile in OI Murine Osteoblasts

**FIGURE 1:**
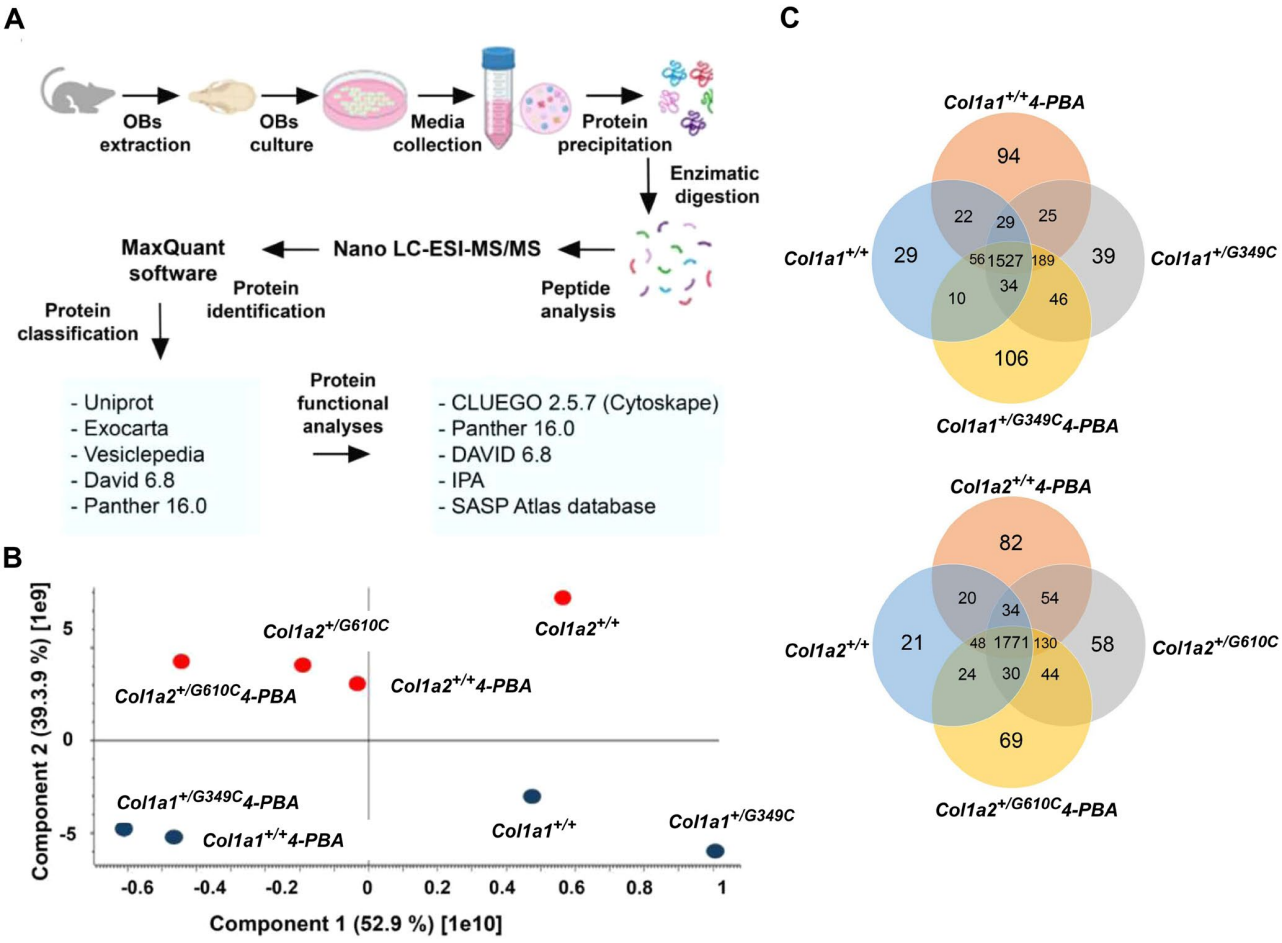
Proteomic analysis of the osteoblast secretome in the *Col1a1* ^*+/G349C*^ and *Col1a2* ^*+/G610C*^ OI models in absence and in presence of 4‐PBA. (A) Overview of the bottom‐up proteomic workflow. Primary osteoblasts were isolated from *Col1a1* ^*+/+*^, *Col1a1* ^*+/G349C*^, *Col1a2* ^*+/+*^ and *Col1a2* ^*+/G610C*^ mice calvaria. Cells were cultured with or without 4‐PBA and medium was collected. Upon protein extraction, enzymatic digestion and purification on C18 resin tips, the peptides were analysed in the first MS scan and peptide fragmentation patterns were obtained in the second MS scan (MS/MS). Biological triplicates for each experiment were performed. The data were processed and analysed using the Max Quant and Perseus platforms. (B) Principal Component Analysis (PCA) of all data sets (*Col1a1* ^*+/+*^, *Col1a1* ^*+/+*^ 4‐PBA, *Col1a1* ^*+/G349C*^, *Col1a1* ^*+/G349C*^ 4‐PBA, *Col1a2* ^*+/+*^, *Col1a2* ^*+/+*^ 4‐PBA, *Col1a2* ^*+/G610C*^, *Col1a2* ^*+/G610C*^ 4‐PBA). (C) Graphical representation of the proteins commonly expressed in the various groups and proteins exclusively present in each data set as determined by one‐way ANOVA analysis followed by post hoc tests with the Bonferroni's correction (p value ≤ 0.05).

**FIGURE 2:**
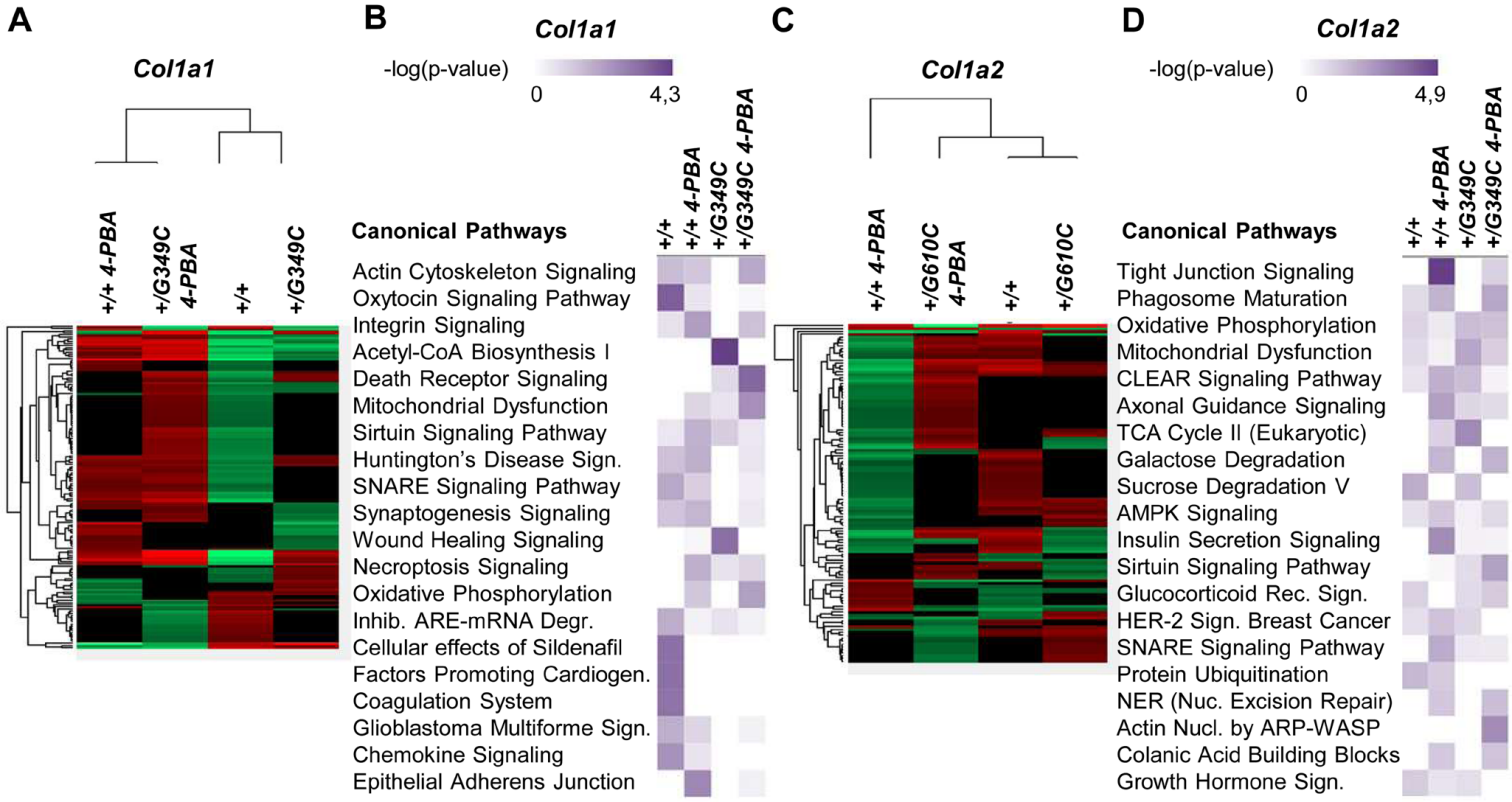
Osteoblast secretome profiles in the *Col1a1* ^*+/G349C*^ and *Col1a2* ^*+/G610C*^ OI models in absence and in presence of 4‐PBA. (A, C) Heat map of the *Col1a1* ^*+/+*^, *Col1a1* ^*+/+*^ 4‐PBA, *Col1a1* ^*+/G349C*^, *Col1a1* ^*+/G349C*^ 4‐PBA secretome and of *Col1a2* ^*+/+*^, *Col1a2* ^*+/+*^ 4‐PBA, *Col1a2* ^*+/G610C*^, *Col1a2* ^*+/G610C*^ 4‐PBA secretome, respectively. The analyses were conducted by a shotgun label free proteomic approach. (B, D) Proteins found to be significantly different across conditions by ANOVA were subjected to bioinformatic analysis using Ingenuity Pathway Analysis (IPA) to explore potential biological pathways and functions. For clarity, the figure reports only 20 of the canonical pathways enriched in the four data sets. The top 5 canonical pathways identified in each data set are shown in Tables 3 and 4. No additional threshold was applied for pathway selection. Biological triplicates for each experiment were performed.

Given the relevance of the secreted factors for OB activity and cellular cross talk, the OB secretome of *Col1a1* ^+/G349C^ and *Col1a2* ^+/G610C^ models, and of their respective controls, was analysed by a label free shotgun proteomic approach (Figure 1A). To unravel the underlying mechanism of the beneficial effect of 4‐PBA on cell homeostasis, the secretome profile was also evaluated on the same models following the cell incubation with the chemical chaperone. To this end, based on previous evidence, we cultured primary murine osteoblasts using a culture protocol optimized to detect ER stress activation in mutant samples. A principal component analysis (PCA) was carried out by grouping quantitative data related to each genotype and condition (untreated and 4‐PBA treated), which indicates a differential clustering of the 8 protein data sets with a clear separation among the *Col1a1* ^+/G349C^ and *Col1a2* ^+/G610C^ data (Figure 1B). The Pearson correlation analyses performed on 3 biological replicates in each model indicated strong data consistency (Figure S1). Uniprot, ExoCarta, Vesiclepedia databases, David and Panther, Secretome P and Signal P were employed to classify the proteins as secreted or not secreted in each data set (Table 1). Only the proteins labelled as secreted, which accounts for more than 90% of the total, were further analysed by various bioinformatic tools to identify pathways and processes modified by the disease and involved in 4‐PBA treatment response. Figure 1C shows the Venn diagram of the proteins differently expressed in the various groups (either commonly expressed or exclusively expressed in only one of them) upon one‐way ANOVA analysis followed by post hoc tests with the Bonferroni's correction (*p*‐value ≤ 0.05). The proteins exclusively expressed in each sample (Tables S1–S8) were further analysed by Panther and DAVID and by IPA (Table 2 and Figure 2) to cluster them into functional groups, according to their involvement in the main networks and pathways. The analysis suggested that proteins involved in cytoskeleton organization and cell adhesion, and metabolism are mainly present in *Col1a1* ^+/G349C^ and *Col1a2* ^+/G610C^ OB secretome. Taking into account the top five canonical pathways identified in each dataset, *Col1a1* ^*+/G349C*^ mice showed enrichment in acetyl‐CoA biosynthesis I, cholesterol biosynthesis, D‐mannose degradation, glycine degradation (linked to creatine biosynthesis), and wound healing signalling pathway (Table 2). In contrast, *Col1a2* ^*+/G610C*^ OBs were enriched for the TCA cycle, the 2‐ketoglutarate dehydrogenase complex, and serine and glycine biosynthesis I (Table 2). Moreover, bioinformatic analysis revealed that several pathways that were previously enriched in the mutant cells were no longer prominent after 4‐PBA incubation, suggesting a partial restoration of physiological cellular function (Table 2). Nevertheless, 4‐PBA upregulated distinct pathways in the two wild‐type models such as epithelial adherens junction signalling, chronic myeloid leukaemia signalling, UDP‐D‐xylose and UDP‐D‐glucuronate biosynthesis in *Col1a1* ^+/+^ and UDP‐N‐acetyl‐D‐galactosamine biosynthesis I, sirtuin signalling, phagosome maturation, actin nucleation by ARP‐WASP complex and integrin signalling in *Col1a2* ^+/+^ (Table 2).

**TABLE 1:** Total and secreted proteins identified in *Col1a1* ^+/+^, *Col1a1* ^*+/G349C*^, *Col1a2* ^*+/+*^ and *Col1a2* ^*+/G610C*^ OB secretome with and without 4‐PBA incubation.

Dataset	*n*° total proteins	*n*° secreted proteins	% secreted proteins
*Col1a1* ^*+/+*^	1723	1648	95.65
*Col1a1* ^*+/+*^ 4‐PBA	2096	1948	92.94
*Col1a1* ^*+/G349C*^	1905	1791	94.02
*Col1a1* ^*+/G349C*^ 4‐PBA	2122	1964	92.55
*Col1a2* ^*+/+*^	1965	1843	93.79
*Col1a2* ^*+/+*^ 4‐PBA	2188	2033	92.92
*Col1a2* ^*+/G610C*^	2138	1996	93.36
*Col1a2* ^*+/G610C*^ 4‐PBA	2165	2017	93.16

*Note:* In each data set the proteins were classified as secreted or not based on Uniprot, ExoCarta, Vesiclepedia databases, David 6.8 (Fischer's exact test *p* ≤ 0.05 and at least 2 counts) and Panther classification 16.0 (Fischer's exact test *p* ≤ 0.05 and at least 2 counts), Secretome P and Signal P. Only proteins present in 3 out of 3 biological repeats were considered.

**TABLE 2:** Bioinformatic analysis by Ingenuity Pathway Analysis (IPA) software of the proteins exclusively expressed in *Col1a1* ^+/+^, *Col1a1* ^*+/G349C*^, *Col1a2* ^*+/+*^and *Col1a2* ^*+/G610C*^ OB secretome with and without 4‐PBA treatment.

Canonical pathways	*Col1a1* ^+/+^	*Col1a1* ^+/+^ 4‐PBA	*Col1a1* ^+/G349C^	*Col1a1* ^+/G349C^ 4‐PBA	*Col1a2* ^*+/+*^	*Col1a2* ^*+/+*^ 4‐PBA	*Col1a2* ^*+/G610C*^	*Col1a2* ^*+/G610C*^ 4‐PBA
Mitochondrial dysfunction		0.949	0.641	**2.521**	0.939	0.402	**2.281**	1.241
Oxidative phosphorylation		1.271		**2.163**	1.118	0.555	1.660	1.581
TCA cycle II (eukaryotic)						1.167	**2.955**	
Acetyl‐CoA biosynthesis I			**4.335**					
Superpathway of cholesterol biosynthesis			**3.057**			1.088		
Lanosterol biosynthesis						**2.533**		
Sucrose degradation V (mammalian)					**2.107**		1.650	
Glycolysis I					**1.720**			
Pentose phosphate pathway (oxidative branch)				**1.763**				
D‐mannose degradation			**2.821**					
2‐ketoglutarate dehydrogenase complex							**2.087**	
Superpathway of serine and glycine biosynthesis I							**1.990**	
Glycine degradation (creatine biosynthesis)			**2.521**					
Glycine biosynthesis I							**2.387**	
Asparagine degradation I						**2.232**		
UDP‐N‐acetyl‐D‐galactosamine biosynthesis I								**2.645**
Phagosome maturation	0.797	1.001		0.975	0.969	1.940		**2.250**
Axonal guidance signalling	0.976	0.613	0.748	0.993		**2.421**	1.062	0.966
Actin cytoskeleton signalling	1.537	1.306		**1.975**		0.288	0.401	0.369
Epithelial Adherens junction signalling		**2.713**		0.374		0.431		0.523
Tight junction signalling	0.752	0.346		0.334		**4.854**		1.211
Insulin secretion signalling pathway	0.587	0.633	1.196	1.155		**2.904**	0.365	0.334
Integrin signalling	0.682	**2.240**		1.417		0.331	0.449	1.904
Sirtuin signalling pathway	0.562	1.774	1.145	0.568		0.238	0.918	**2.367**
Death receptor signalling			0.869	**3.450**		0.609	1.778	
Protein ubiquitination pathway	0.584	0.628	1.190	1.148	**1.794**	1.326		
Oxytocin signalling pathway	**3.633**	0.612		0.202		0.698		0.324
Actin nucleation by ARP‐WASP complex		**2.421**						**2.909**
Cellular effects of sildenafil (viagra)	**3.244**					2.008		
Factors promoting cardiogenesis	**3.219**					1.129		
Wound healing signalling pathway		0.242	**3.260**				0.391	0.360
IL‐4 signalling		0.571			**2.706**			
Coagulation system	**3.177**							
Coronavirus replication pathway				0.838				**2.330**
Intrinsic prothrombin activation pathway	**3.019**							
Chronic myeloid leukaemia signalling		**2.251**						
UDP‐D‐xylose and UDP‐D‐glucuronate biosynthesis		**2.174**						
Apelin liver signalling pathway					**1.736**			

*Note:* Bioinformatic analyses were carried out to cluster enriched GO terms, networks and pathways within the proteins exclusively expressed in each data set upon an ANOVA analysis followed by post hoc tests with the Bonferroni's correction (*p*‐value ≤ 0.05). Only proteins consistently present and quantified across all three biological replicates were considered. Functional grouping was based on Fischer's exact test (*p* ≤ 0.05). The Table reports in bold the top 5 canonical pathways identified in each data set. No additional threshold was applied for pathway selection. Values are expressed in ‐Log *p*‐value. The pathways “reverted” by the 4‐PBA treatment are in grey.

To further characterize genotype and 4‐PBA effect in the different models, a deep analysis of similarities and differences in the secretome was carried out by bioinformatic software considering all the proteins differentially expressed. The following comparisons were evaluated: *Col1a1* ^+/G349C^ versus *Col1a1* ^+/+^, *Col1a1* ^+/G349C^ 4‐PBA versus *Col1a1* ^+/+^ 4‐PBA, *Col1a1* ^+/G349C^ 4‐PBA versus *Col1a1* ^+/+^, *Col1a2* ^+/G610C^ versus *Col1a2* ^+/+^, *Col1a2* ^+/G610C^ 4‐PBA versus *Col1a2* ^+/+^ 4‐PBA; *Col1a2* ^+/G610C^ 4‐PBA versus *Col1a2* ^+/+^(Tables S9–S14). To highlight possible differences among the models with mutations in the collagen I α1 and α2 chains, the comparisons *Col1a1* ^*+/+*^ versus *Col1a2* ^*+/+*^, *Col1a1* ^*+/+*^ 4‐PBA versus *Col1a2* ^*+/+*^ 4‐PBA, *Col1a1* ^*+/G349C*^ versus *Col1a2* ^*+/G610C*^, *Col1a1* ^*+/G349C*^ 4‐PBA versus *Col1a2* ^*+/G610C*^ 4‐PBA were assessed (Tables S15–S18). The results obtained by Panther on the proteins listed in Tables S9–S14 are reported for the *Col1a1* ^*+/G349C*^ model in Table 3 and for the *Col1a2* ^*+/G610C*^ model in Table 4. Focusing on the comparison between the *Col1a1* ^+/G349C^ and *Col1a2* ^+/G610C^ mice (Tables S3 and S7; S15–S18), the bioinformatic analysis by Panther and Cluego is reported in Figures S2 and S3; Tables S19 and S20, respectively. Proteins common to both datasets were predominantly associated with energy metabolism (DLAT, NDUFS1, ADH1), protein synthesis and translation (EEF1e1, RPL36a), and cytoskeletal organization (MYO1b, ARPC1a, KRT35, KRT42) (Tables S3, S7, and S15–S18). Proteins unique to *Col1a1* ^+/G349C^ (Table S17) clustered within pathways related to mitochondrial and carbohydrate metabolism (ACAT1, DLAT, DECR1, NDUFS1, HADH, ALDOC, SUCLA2, MPI, ADI1, and DAK), to cellular stress responses (SQSTM1, NDRG1, TRAP1) and to vesicular trafficking and protein turnover pathways (UCHL1, PSMC1, COPG2, RAB1b, TMED10, and SEC16a), the latter pointing to active endomembrane dynamics and secretory function. For what concerns proteins uniquely expressed or with an increased expression in the *Col1a2* ^+/G610C^ model (Tables S7 and S17), COX5a, SDHA, OGDH, SHMT2, KHK, PCK2, and ACO1 clustered within pathways involved in mitochondrial energy metabolism and amino acid catabolism; ABCE1, EIF4A3, EIF4EBP2, SF3B4, PRPF8, and NOP58 participate in RNA processing and protein translation, suggesting elevated biosynthetic activity. Additionally, DNAJC10, NCSTN, HSPBP1, and USP15 are involved in protein folding and ER‐associated degradation pathways, VPS4b, RAB21, VAMP7, TMED7, SNAP29, and SEC61b are involved in vesicular trafficking and secretion, while BCL2l13, PARP, and WIPI1 belong to autophagy and apoptosis regulation pathways. Focusing on the 4‐PBA effect, an increased presence of proteins involved in translation, interferon signaling, apoptotic protein cleavage, EGFR, MET, VEGF signaling, and RHOG GTPase cycle was found in the secretome of treated *Col1a1* ^*+/G349C*^ osteoblasts (Table 3). Pathways related to fibril coat formation, platelet degranulation, regulation of insulin‐like growth factor (IGF) transport by IGFBPs, and blood coagulation were instead decreased (Table 3). The protein dataset derived from the secretome of 4‐PBA incubated *Col1a2* ^+/G610C^ osteoblasts points to increased ion homeostasis, signaling by RHO, Miro, and RHOBTB3 GTPases, mRNA splicing, asparagine N‐linked glycosylation, and reduced post translational modification (PTM) pathways (Table 4). Innate immune activation and membrane trafficking were similarly increased in both models upon chaperone incubation (Tables 3 and 4).

**TABLE 3:** Bioinformatic analysis by Panther software of the proteins differentially or exclusively expressed in the comparison *Col1a1* ^+/G349C^ 4‐PBA versus *Col1a1* ^+/+^.

Exclusively expressed or increased in *Col1a1* ^*+/G349C*^ 4‐PBA				
Panther reactome pathways	Counts	Fold Enrichment	Raw *p*	FDR
Translation	18	4.33	5.29E‐07	5.29E‐05
Interferon signalling	7	5.42	4.93E‐04	2.09E‐02
Apoptotic cleavage of cellular proteins	5	7.13	1.04E‐03	3.77E‐02
Signalling by EGFR	6	7.07	3.44E‐04	1.67E‐02
RHOG GTPase cycle	9	6.59	1.94E‐05	1.18E‐03
Signalling by MET	7	4.86	8.94E‐04	3.38E‐02
Signalling by VEGF	8	4.33	7.90E‐04	3.20E‐02
Innate immune system	44	2.28	9.56E‐07	8.56E‐05
Membrane trafficking	22	2.13	1.26E‐03	4.46E‐02

Decreased in *Col1a1* ^*+/G349C*^ 4‐PBA or only expressed in *Col1a1* ^*+/+*^				
Panther reactome pathways	Counts	Fold enrichment	Raw *p*	FDR
Formation of fibrin clot	5	32.16	8.55E‐07	3.64E‐04
Platelet degranulation	6	12.02	1.41E‐05	4.79E‐03
Regulation of insulin‐like growth factor (IGF) transport and uptake by insulin‐like growth factor binding proteins (IGFBPs)	8	16.03	6.00E‐08	3.40E‐05

Panther pathways	Counts	Fold enrichment	Raw *p*	FDR
Blood coagulation	5	23.5	3.56E‐06	5.73E‐04

*Note:* Proteins were considered differentially expressed in the comparison if they showed significant *t*‐test difference (FDR ≤ 0.05) or were expressed exclusively in one condition. Only proteins consistently present and quantified across all three biological replicates were considered. If any Panther pathways enrichment was found, the data were processed by Panther Reactome to find Reactome GO and pathways enrichment. Functional grouping was based on Fischer's exact test (*p* ≤ 0.05) and at least 2 genes count.

**TABLE 4:** Bioinformatic analysis by Panther software of the proteins differentially or exclusively expressed in the comparison *Col1a2* ^*+*/G610C^ 4‐PBA versus *Col1a2* ^*+*/+^.

Exclusively expressed or increased in *Col1a2* ^*+/G610C*^ 4‐PBA				
Panther reactome pathways	Counts	Fold enrichment	Raw *p*	FDR
Ion homeostasis	5	8.83	3.72E‐04	3.95E‐02
Signalling by Rho GTPases, Miro GTPases and RHOBTB3	24	3.12	1.38E‐06	3.92E‐04
mRNA Splicing—major pathway	13	6.38	2.91E‐07	1.65E‐04
Asparagine N‐linked glycosylation	12	4.05	6.56E‐05	1.12E‐02
Innate immune system	25	2.11	5.01E‐04	4.48E‐02
Membrane trafficking	17	2.69	2.86E‐04	3.48E‐02

Decreased in *Col1a2* ^*+/G610C*^ 4‐PBA or only expressed in *Col1a2* ^*+/+*^				
Panther reactome pathways	Counts	Fold enrichment	Raw *p*	FDR
Post‐translational protein modification	15	3.28	4.44E‐05	2.51E‐02

*Note:* Proteins were considered differentially expressed in the comparison if they showed a significant *t*‐test difference (FDR ≤ 0.05) or were expressed exclusively in one condition. Only proteins consistently present and quantified across all three biological replicates were considered. If any Panther pathways enrichment was found, the data were processed by Panther Reactome to find Reactome GO and pathways enrichment. Functional grouping was based on Fischer's exact test (*p* ≤ 0.05) and at least 2 genes count.

### 3.2 Senescence‐Associated Proteins Are Enriched in the Secretome of *Col1a1^+/G349C^* and *Col1a2* ^*+/G610C*^ Osteoblasts and Attenuated by 4‐PBA Treatment

**FIGURE 3:**
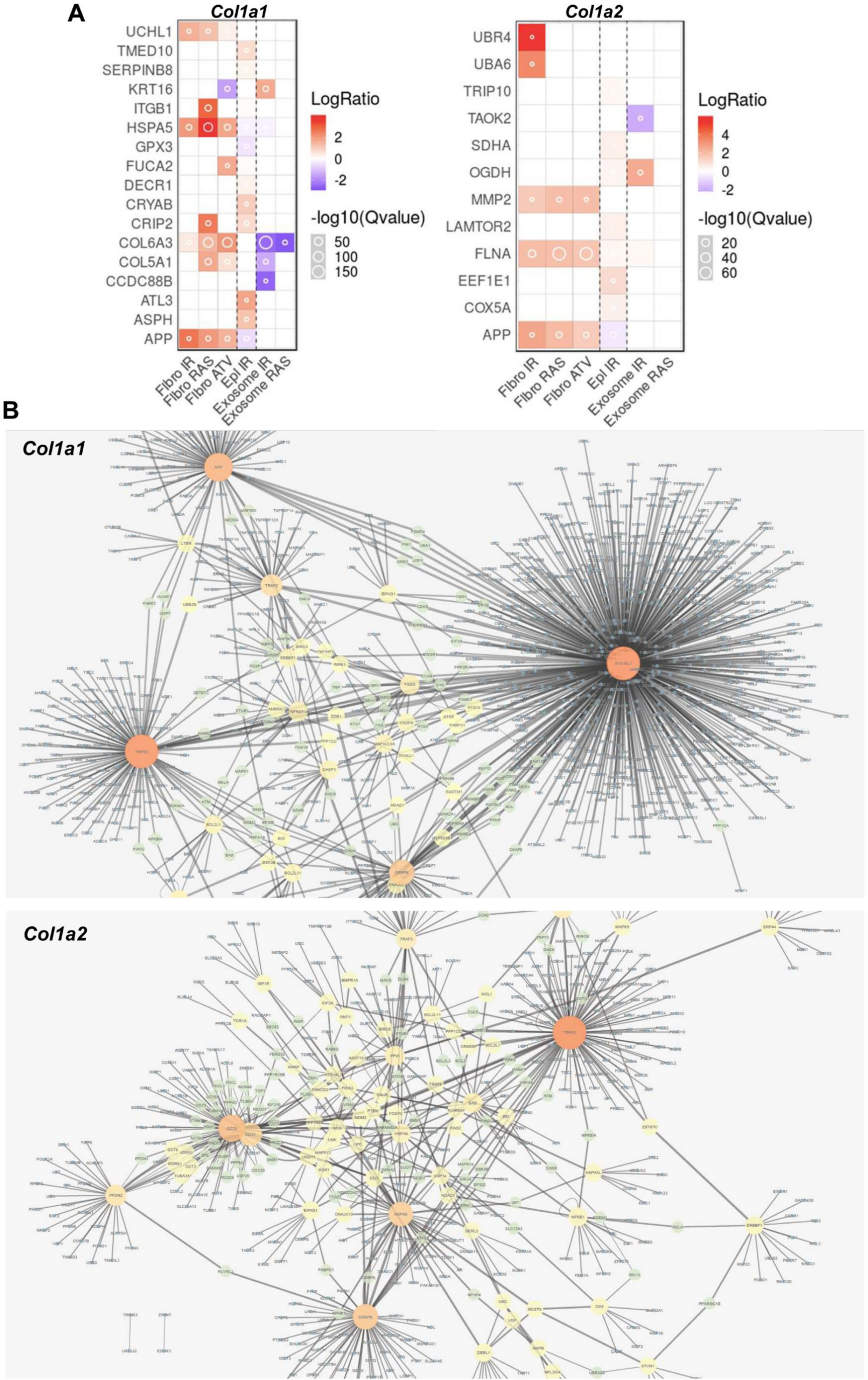
Senescence associated secretory phenotype proteins and senescence associated genes in *Col1a1* ^*+/G349C*^ and *Col1a2* ^*+/G610C*^ OI osteoblasts. (A) Functional proteomic analysis of secretome data was performed to identify secreted proteins associated with the senescence‐associated secretory phenotype (SASP), using the SASP Atlas database. Fibro IR: Senescence in fibroblasts induced by X‐irradiation. Fibro RAS: Senescence in fibroblasts induced by RAS overexpression. Fibro ATV: Senescence in fibroblasts induced by atazanavir (ATV) treatment. EpI IR: Senescence in epithelial cells induced by X‐irradiation. Exosome IR: Exosome SASP of senescent fibroblasts induced by X‐irradiation. Exosome RAS: Exosome SASP of senescent fibroblasts induced by RAS overexpression. (B) Bioinformatic analyses of the qPCR‐based transcriptome in *Col1a1* ^*+/G349C*^ and *Col1a2* ^*+/G610C*^ OBs revealed the presence of hub genes, including *P53* in both models and *App* in *Col1a1* ^*+/G349C*^ mice. The high‐resolution file enabling detailed zooming, allowing the names of all proteins to be clearly visualized, is reported in Figure S4 for *Col1a1* ^*+/G349C*^ mice and Figure S5 for *Col1a2* ^*+/G610C*^. Biological triplicates for each experiment were performed.

The presence of SASP‐related proteins in *Col1a1* ^+/G349C^ secretome, such as αβ‐crystallin (CRYAB), olfactomedin like 3 (OLFML3), serpin B8 (SERPINB8), and α‐L‐fucosidase 2 (FUCA2) (Table S3), and in *Col1a2* ^+/G610C^ secretome, such as ubiquitin protein ligase E3 component N‐recognin 4 (UBR4), matrix metalloproteinase 2 (MMP2), and especially lysosomal‐β‐galactosidase (GLB1L) (Table S7), suggests a senescence‐like phenotype in mutant OBs, likely as a response to ER stress due to misfolded collagen retention [50]. Importantly, proteomic analyses also identified amyloid‐precursor protein (APP) in *Col1a2* ^+/G610C^ OBs (Table S17), which according to recent findings is also involved in senescence [51]. Moreover, 17 proteins found exclusively in *Col1a1* ^+/G349C^ and 12 proteins found exclusively in *Col1a2* ^+/G610C^ OBs secretome are included in senescence‐associated secretory phenotype through the SASP Atlas (Figure 3A), a comprehensive proteomic database of soluble proteins and exosomal cargo SASP factors originating from multiple senescence inducers and cell types [40]. Notably, incubation with 4‐PBA did not result in the detection of senescence‐associated markers in the secretome of either model.

### 3.3 Transcriptomic and Cellular Assay Confirmed Senescence Activation and Its Rescue by 4‐PBA Incubation

**FIGURE 4:**
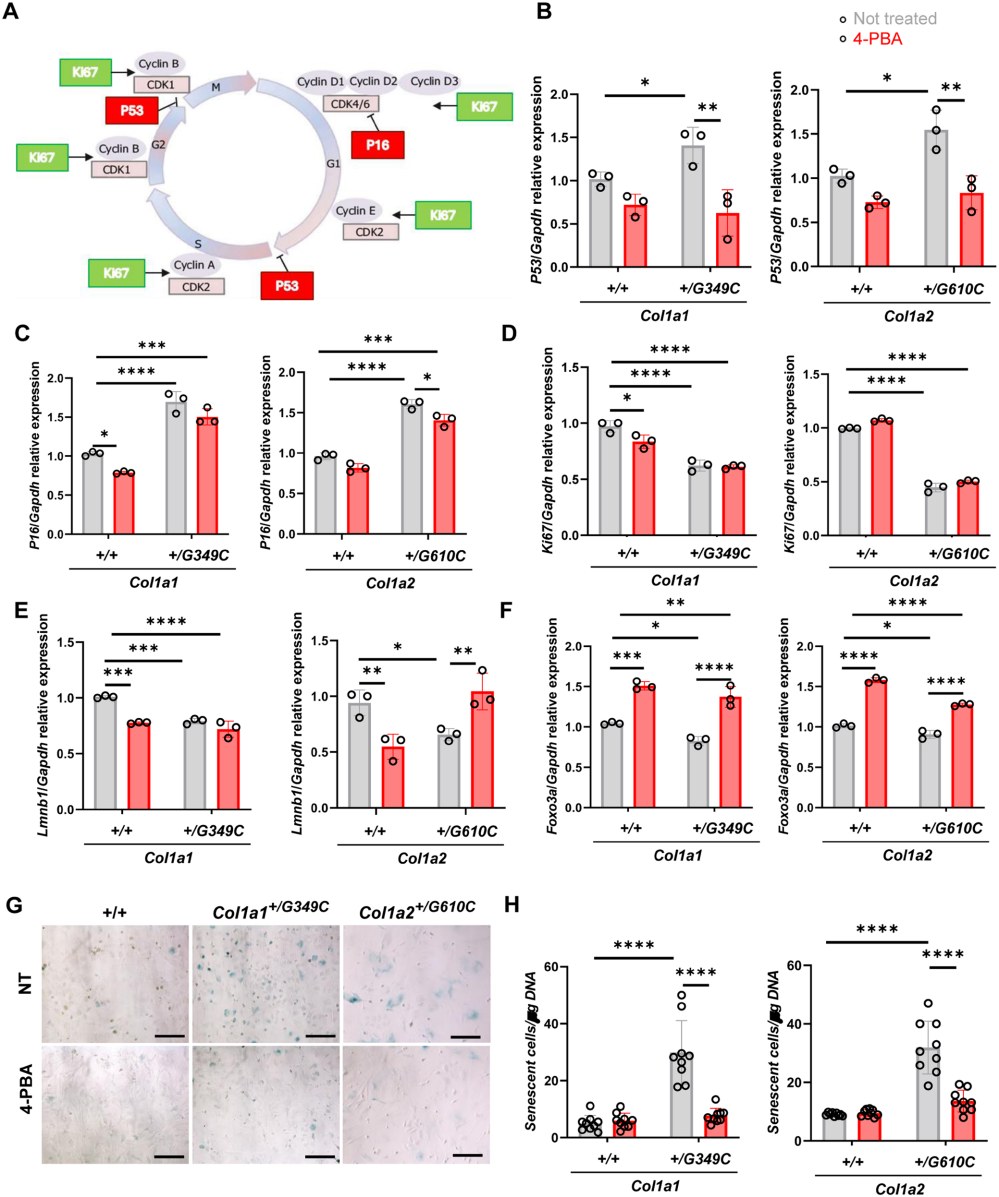
Senescence is attenuated by 4‐PBA in *Col1a1* ^+/G349C^ and *Col1a2* ^*+/G610C*^ OBs. (A) Scheme of the main genes involved in the regulation of cell cycle progression. Real time PCR analyses of *P53* (B), *P16* (C), *Ki67* (D), *Lmnb1* (E), and *Foxo3* (F) expression in *Col1a1* ^+/G349C^ and *Col1a2* ^*+/G610C*^ OBs in absence or presence of 4‐PBA. (G, H) SA‐β‐gal staining and quantification of senescent cells in *Col1a1* ^+/G349C^ and *Col1a2* ^*+/G610C*^ OBs in absence or presence of 4‐PBA. Biological triplicates for each experiment were performed. *p < 0.05, **p < 0.01, ***p < 0.001, ****p < 0.0001.

Bioinformatic analyses of the qPCR‐based transcriptome (Figure 3B; Figures S4 and S5 for the high‐resolution file enabling detailed zooming) revealed in both mutant OBs the presence of a few hub genes, including *P53*, the major regulator of the cell cycle that drives the activation of cyclin‐dependent kinase inhibitors and ultimately leads to cell cycle arrest. Furthermore, the hub gene *App*, a regulator of the TNF‐α signalling which in turn modulates *P53* expression and thus senescence, was found in *Col1a1* ^+/G349C^ OBs, strengthening the proteomic data. NF‐κB also emerged as a central hub in both models. qPCR analysis performed on independent samples collected from cells grown in identical conditions confirmed in both models the upregulation of *P53* and of *P16* expression, among the most well‐established senescence markers known to arrest the cell cycle by blocking progression through G1/S (Figure 4A). 4‐PBA incubation significantly lowered *P53* in both models and *P16* expression in *Col1a2* ^+/G610C^ (Figure 4B,C). Consistently, *Ki67*, a nuclear protein marker of proliferating cells (Figure 4D), and laminin b1 (*Lmnb1*) (Figure 4E), a marker for nuclear morphology that is often lost in senescent cells, were found significantly reduced in both mutant OBs. The latter was rescued by 4‐PBA administration in *Col1a2* ^+/G610C^. Interestingly, qPCR data on samples obtained following 4‐PBA incubation showed the upregulation of forkhead box O3 (*Foxo3*), known to activate the expression of genes related to protein turnover, supporting the stimulation of an adaptative response to reverse senescence (Figure 4F). Lastly, β‐galactosidase activity assay revealed a higher number of mutant cells positive for SA‐β‐Gal staining compared to control cells that was reduced by the chaperone administration (Figure 4G,H), confirming the increased senescence in both *Col1a1* ^+/G349C^ and *Col1a2* ^+/G610C^ models and the positive effect of 4‐PBA.

### 3.4 Cytoskeletal and Cellular Adhesion Changes in *Col1a1* ^+/G349C^ and *Col1a2* ^+/G610C^ Osteoblasts Are Modulated by 4‐PBA

Senescence is known to be associated with cytoskeletal abnormality, and indeed proteins involved in actin cytoskeleton crosslinking (Cdc42‐interacting protein 4, Sorbin and SH3 domain‐containing protein 2, SH3 domain‐binding protein 1) and cell adhesion (PARVB, SEMA3B, SEMA3C) were uniquely found in *Col1a2* ^+/G610C^ derived OB secretome (Table S7). Similarly, in *Col1a1* ^+/G349C^ secretome, crystallin alpha B (CRYAB), that interacts with actin and enhances microtubule stability [52], was found together with some of the main adherents junction proteins (PCDHGC3, SERPINB8, PLXND1) or proteins that contribute to cell adhesion (NPNT, OLFML3) (Table S3).

**FIGURE 5:**
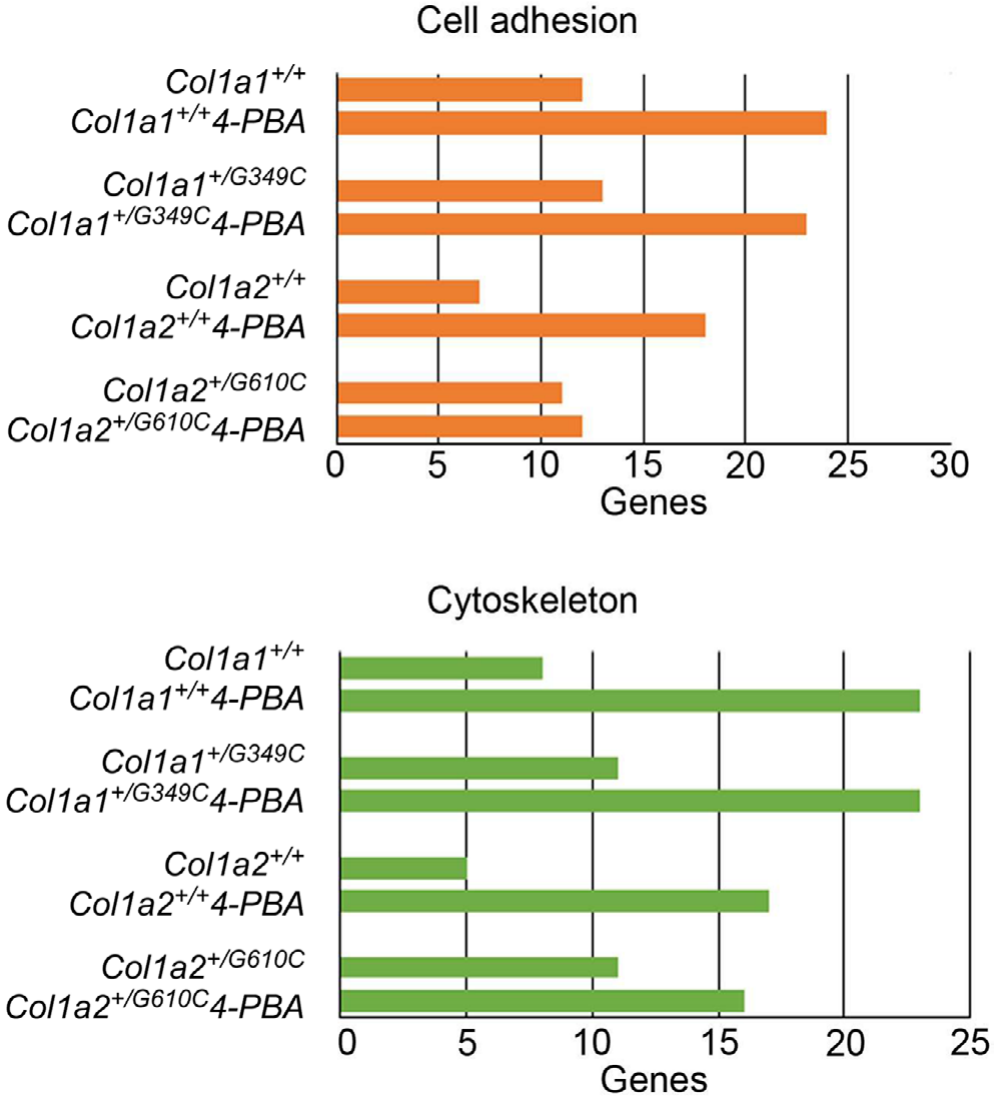
Focus on cytoskeleton and cell adhesion by Ingenuity Pathway Analysis (IPA) software of the proteins exclusively expressed in *Col1a1* ^*+/+*^, *Col1a1* ^*+/+*^ 4‐PBA, *Col1a1* ^*+/G349C*^, *Col1a1* ^*+/G349C*^ 4‐PBA, *Col1a2* ^*+/+*^, *Col1a2* ^*+/+*^ 4‐PBA, *Col1a2* ^*+/G610C*^ and *Col1a2* ^*+/G610C*^ 4‐PBA OB secretome. Bioinformatic analyses were carried out based on cytoskeleton and cell adhesion keywords, within the proteins exclusively expressed upon an ANOVA analysis followed by post hoc tests with the Bonferroni's correction (*p* ≤ 0.05). Biological triplicates for each experiment were performed.

Other factors regulating cell adhesion or cytoskeletal structures and that control osteoblast function and bone formation (CAPZA1, ADD1, TUBB2B, CEP95, DST, SVIL, XIRP2, NCK1, NDRG1, BAIAP2, RHOG) were found upon 4‐PBA incubation in *Col1a2* ^+/G610C^ OBs (Table S8). Consistently, several regulators of actin filament dynamics and stimulators of actin bundling (TMSB10, SVIL, CALD1, GSN, MAP6, PPP4C, SNTB2, PLEK, TES) were detected in *Col1a1* ^+/G349C^ OBs upon 4‐PBA administration (Table S4). These findings prompted us to focus on the cytoskeleton and cellular adhesion by IPA. The results reported in Table 5 and in Figure 5 clearly confirm that, among the proteins exclusively expressed in the 8 data sets, many are related to these two keywords and impact different pathways. In the *Col1a1* ^*+/G349C*^ model, the secretome profile is enriched in ECM and structural proteins such as COL5A1, COL6A3, KRT16 and NPNT (Table 5), suggesting a strong impact on matrix composition and tissue architecture. Proteins such as BOC, PLXND1 and SLIT3 (Table 5) suggest altered axon guidance‐related pathways, which may be repurposed in osteoblast communication or migration [53]. In the *Col1a2* ^*+/G610C*^ model, the presence of several regulators of intracellular trafficking such as CCM2, STXBP2, TRIP10, and UBA6 (Table 5) points to a more prominent involvement of vesicular transport.

**TABLE 5:** Focus on cytoskeleton and cell adhesion by Ingenuity Pathway Analysis (IPA) software of the proteins exclusively expressed in the secretome of ***Col1a1* ^*+/+*^**, ***Col1a1* ^*+/+*^** 4‐PBA, ***Col1a1* ^*+/G349C*^**, ***Col1a1* ^*+/G349C*^** 4‐PBA, and in the secretome of ***Col1a2* ^*+/+*^**, ***Col1a2* ^*+/+*^** 4‐PBA, ***Col1a2* ^*+/G610C*^**, ***Col1a2* ^*+/G610C*^** 4‐PBA osteoblasts.

	*Col1a1* ^*+/+*^		*Col1a1* ^*+/G349C*^		*Col1a1* ^*+/+*^ 4‐PBA		*Col1a1* ^*+/G349C*^ 4‐PBA	
	‐log(*p*)	Proteins	‐log(*p*)	Proteins	‐log(*p*)	Proteins	‐log(*p*)	Proteins
Cell adhesion	2.66E+00	ANG,CDH13,F5,FZD1, HYAL1,ITIH5,KLKB1,MAPK15,MYH6,PLCB4,PTPRS,SNCG	1.8	BOC,COL5A1,COL6A3,CXCL6,GMFG,KRT16,LY6A (includes others),NPNT,PCDHGC3,PDHA1,PLXND1,TNFSF12,UCHL1	1.7	ADD1,ATG7,CCM2,CKM,CRK,DNM3,FLOT1,FNTA,GIPC1,JAG1,JUP,LY6E,MACROH2A1,MGAT1,NRAS,NSF,PLOD2,PRKAB1,PRRX1,PSMD4,RHOG,SNU13,TNS3,WAS	1.28	CALD1,CD151,CDK2,CFDP1,DIAPH1,DNM1,DOCK1,EIF4EBP2,ERBIN,GAS2,GSN,HAPLN1,HSPB7,MDGA1,PARP1,PARVB,PTN,QRFPR,SGCE,SLIT2,TGFB1I1,TUBB2B,VTI1B
Cytoskeleton	9.60E‐01	ANG,CDH13,FZD1,MAPK15,MYH6,PLCB4,PPP1R12b,PTPRS	1.2	BOC,COL5A1,COL6A3,CRYAB,CXCL6,GMFG,KRT16,P3H1,PLXND1,SLIT3,UCHL1	1.6	ABR,ADD1,ATG7,CCM2,CKM,CRK,DNM3,DYNLL1,EPHX2,FLOT1,GDA,JAG1,JUP,KIF2A,NRAS,PQBP1,PRKAB1,RHOG,SNAP29,STRN,SYNE2,TNS3,WAS	1.42	ATG5,CALD1,DIAPH1,DNM1,DOCK1,EIF4EBP2,ERBIN,GAS2,GSN,HSPB7,MAP6,NCKAP1,NCS1,NCSTN,PARP1,PARVB,PLEK,PTN,PYCARD,QRFPR,SLIT2,SMARCE1,TUBB2B

	Col1a2^+/+^		Col1a2^+/G610C^		*Col1a2* ^*+/+*^ 4‐PBA		*Col1a2* ^*+/G610C*^ 4‐PBA	
Cell adhesion	1.59	CDH10,CXCL16,HLA‐A,IRS1,SFRP4,SLC12A2,UBE3A	0.422	CCM2,CD14,CFDP1, GAS2,LFNG,MMP2,PARVB,SEMA3B,SORBS2,STXBP2,TNFSF12	0.852	CCl6,CD151,CKM,CLIP1, CORO1A,FZD1,GLA,LY6E,MYH11,NAPG,PLCG1,PPP2R5D,PRKACB,SAT2,SLIT2,SPTAN1,TJP1,TRAP1	0.417	ADD1,BAIAP2,DAB2,DRG2,JAG1,LIMS1,NCK1,NSF,PF4,RHOG,TPBG,TUBB2B
Cytoskeleton	0.748	CDH10,EML2,HLA‐A,IRS1,UBE3A	0.474	CCM2,GAS2,MMP2, NEK7,PARVB,SEMA3B,SEMA3C,SH3BP1,SORBS2,TRIP10,UBA6	0.755	ANK2,BAG5,CKM,CLIP1,CORO1A,DYNLL1,EXOC7,FZD1,GALK2,MYH11,PLCG1,PPP2R5D,PRKACB,SEMA7A,SLIT2,SPTAN1,TJP1	1.36	ADD1,BAIAP2,CAPZA1,CRLF3,DAB2,DST,FHL2,JAG1,NCK1,NDRG1,PF4,PQBP1,RHOG,SMARCE1,TPBG,TUBB2B

*Note:* Bioinformatic analyses were carried out based on cytoskeleton and cell adhesion keywords, within the proteins exclusively expressed upon an ANOVA analysis followed by post hoc tests with the Bonferroni's correction (*p*‐value ≤ 0.05). Only proteins consistently present and quantified across all three biological replicates were considered.

Following 4‐PBA incubation, the *Col1a1* ^*+/G349C*^ model shows an enrichment of proteins involved in cytoskeletal organization, vesicle trafficking, and signalling. 4‐PBA induced the upregulation of specific regulators of cytoskeletal organization (CALD1, DIAPH1, DNM1, DOCK1, GAS2, GSN, MAP6, NCKAP1, TUBB2B and PARVB), suggesting a profound remodelling of the cytoskeletal network (Table 5). The increased level of signalling molecules (PTN, SLIT2) and extracellular interaction mediators (CD151, QRFPR, SGCE, ERBIN and TGFB1I1) points to enhanced cell–cell communication and modulation of the microenvironment (Table 5). The presence of vesicle trafficking proteins (VTI1B, NCS1, PLEK) implies a reorganization of intracellular transport while the presence of structural (CFDP1, MDGA1) and extracellular matrix components (HAPLN1) further reinforces the hypothesis of treatment‐driven changes in tissue architecture and cell–matrix interactions (Table 5). An enrichment of proteins involved in cytoskeletal organization (ADD1, BAIAP2, CAPZA1, DST, RHOG, TUBB2B, NCK1) and cell adhesion, such as JAG1, LIMS1, TPBG, FHL2, and DAB2, is found in *Col1a2* ^*+/G610C*^ incubated with 4‐PBA, suggesting remodelling of the cytoskeleton and modulation of cell–cell and cell–matrix interactions upon administration (Table 5). As in the *Col1a1* *^+/G349C^* model, the presence of factors related to vesicle trafficking and membrane dynamics (NSF, NCK1) points to reorganization of intracellular transport (Table 5).

## 4 Discussion

In this in vitro study, by combining secretomic, transcriptomic, and functional analyses, we identified premature cellular senescence as a previously unrecognized consequence of osteoblast dysfunction in OI and demonstrated that 4‐PBA exerts a beneficial effect on osteoblast senescence. Primary osteoblasts were isolated from two murine models of dominant OI, carrying mutations in either the α1 or α2 collagen I chain. Both *Col1a1* ^*+/G349C*^ and *Col1a2* ^*+/G610C*^ osteoblasts exhibited a senescence‐associated secretory phenotype (SASP), accompanied by increased expression of senescence markers. We previously demonstrated that these mutant osteoblasts are characterized by endoplasmic reticulum (ER) stress resulting from intracellular retention of misfolded collagen [6]. Notably, treatment with 4‐PBA, which alleviates ER stress by improving protein folding and secretion, led to a concomitant reduction in senescence markers and SASP factors, supporting a link between ER stress and the activation of a senescent phenotype and indicating that mitigation of ER stress is associated with a decreased osteoblast senescence. Transcriptomic analyses identified P53 and NF*‐κB* as hub genes, supporting a conserved senescence program triggered by mutant collagen‐induced stress. Given the established role of P53 in integrating endoplasmic reticulum stress, DNA damage, and mitochondrial dysfunction [40], our data suggest that intracellular retention of misfolded collagen is sufficient to induce osteoblasts' senescence. While transient senescence may contribute to tissue adaptation, the sustained SASP observed here is likely to impair bone matrix quality and remodelling.

Importantly, our findings provide a conceptual framework for clinical observations of premature aging‐like features in OI patients, including early functional decline and increased fracture susceptibility. A literature review revealed that adult patients with OI experience age‐related symptoms, such as cardiorespiratory issues, vision impairment, increased fracture risk, joint problems, and hearing loss, at an earlier age than typically expected [54] and are also at greater risk of early death, although very few is known about the mechanism underneath these observations [55]. Previous studies in recessive OI models have highlighted a role for senescence‐associated pathways in the manifestation of early aging–like phenotypes [56, 57]. Our data, in line with these findings, further extend them to classical dominant OI models, providing the first in vitro evidence that cellular senescence is associated with endoplasmic reticulum stress caused by intracellular collagen retention.

Beyond shared senescence signatures, we observed distinct metabolic and functional profiles between the two OI models, highlighting the heterogeneity of cellular adaptation to different collagen I mutations. Baseline analyses indicated that *Col1a1* ^*+/G349C*^ osteoblasts preferentially engage lipid metabolism, extracellular matrix remodelling, and tissue repair pathways, whereas *Col1a2* ^*+/G610C*^ cells rely more heavily on oxidative energy production, anabolic amino acid synthesis, and vesicular trafficking. These differences likely reflect mutation‐specific compensatory mechanisms, although we cannot exclude that they may be influenced by the distinct genetic backgrounds of the mouse models, particularly given that the corresponding control strains also differ.

Metabolic alterations in murine OI models have been previously reported. Indeed, *Col1a1* ^*Jrt/+*^ mice display a hypermetabolic phenotype, with increased oxygen consumption, carbon dioxide production, and energy expenditure, alongside sex‐dependent changes in glucose homeostasis and insulin levels, despite reduced adiposity compared to wild‐type controls [58]. Similarly, the severe *oim/oim* mice show altered body composition, enhanced energy expenditure independent of activity, and skeletal muscle mitochondrial dysfunction, suggesting that collagen I mutations can affect energy metabolism beyond bone [59]. Transcriptomic analyses further reveal changes in genes associated with lipid and energy metabolism, while milder models, such as *Col1a2* ^*+/G610C*^, exhibit subtler shifts, highlighting variability depending on mutation severity and genetic background [60].

These observations suggest that metabolic alterations are an underappreciated feature of dominant OI, potentially contributing to muscle weakness, reduced physical performance, and systemic energy imbalance observed in patients [61, 62]. Importantly, our findings on osteoblast senescence and ER stress occur in the context of these systemic metabolic changes, raising the possibility that cellular stress responses in bone may be influenced or exacerbated by broader metabolic perturbations. Future studies integrating bone and systemic metabolism could clarify how these interactions contribute to disease severity and inform the development of context‐aware or personalized therapeutic strategies for OI.

Consistent with the OI heterogeneity, 4‐PBA induced a model‐dependent cellular reprogramming. In *Col1a1* ^*+/G349C*^ osteoblasts, 4‐PBA enhanced mitochondrial efficiency and activated survival and proliferative signalling pathways, including EGFR, MET, and VEGF. In contrast, *Col1a2* ^*+/G610C*^ osteoblasts responded to 4‐PBA with improved lipid biosynthesis, ion homeostasis relevant for mineralization, and strengthened intracellular trafficking. Enhanced mRNA splicing and glycosylation further suggest improved protein synthesis and processing.

Among the several pathways identified in *Col1a2* ^*+/G610C*^ and *Col1a1* ^*+/G349C*^ osteoblasts, the proteomic analyses pointed to alterations of cytoskeleton organization/cell adhesion processes that play essential roles in regulating osteoblastogenesis [63]. The present data align well with previous reports of cytoskeletal anomalies in patients carrying dominant or recessive mutations [64, 65] and in *Col1a1* ^*+/G349C*^ mice with a lethal phenotype, where a bone‐specific down‐regulation of vimentin and an up‐regulation of stathmin were responsible for impairing the formation and organization of microtubules and intermediate filaments, affecting the skeletal structural properties [64]. Treatment with 4‐PBA influenced cytoskeletal reorganization and cell adhesion. This effect is likely mediated either by the restoration of cellular homeostasis or by a direct impact of the drug on cytoskeletal protein folding and structure.

While this study provides mechanistic insight, the in vitro model cannot fully replicate the structural, mechanical, and systemic complexity of bone in vivo. Consequently, the long‐term functional impact of osteoblast senescence on bone remodelling, matrix quality, and fracture susceptibility remains open for further investigation. Future studies will also be needed to evaluate whether targeting senescence in vivo can provide sustained therapeutic benefit. The mutation‐ and/or genetic background–specific responses observed here further emphasize the need for context‐aware, potentially personalized therapeutic strategies in OI, highlighting the importance of disease‐relevant models for dissecting complex cellular stress responses and evaluating interventions in rare skeletal disorders.

## Author Contributions

Conceptualization: A.F., R.B. and G.T.; data curation: E.M. and R.B.; formal analysis: E.M., G.T., V.I., R.B. and A.F.; funding acquisition: A.F. and R.B.; investigation: E.M., R.B., E.P., A.S. and N.G.; methodology: A.S. and E.P.; project administration: R.B. and E.M.; resources: A.F., G.T. and V.I.; software: E.M.,V.I. and G.T.; supervision: A.F. and G.T.; validation: R.B., E.M., A.F. and G.T.; writing original draft: R.B., E.M., A.F. and G.T.; writing – review and editing: all authors.

## Funding

This work was supported by Ministero dell'Università e della Ricerca, 20223C8C5B, 2022ERWJKZ.

## Conflicts of Interest

The authors declare no conflicts of interest.

## Supporting information


**Figure S1:** (A, B) Scatter plots of the correlations between the three biological replicates in all the comparisons.
**Figure S2:** Bioinformatic analysis by Cluego of the proteins differentially or exclusively expressed in *Col1a1* ^*+/+*^ versus *Col1a2* ^*+/+*^, *Col1a1* ^*+/+*^ 4‐PBA versus *Col1a2* ^*+/+*^ 4‐PBA, *Col1a1* ^*+/G349C*^ versus *Col1a2* ^*+/G610C*^, *Col1a1* ^*+/G349C*^ 4‐PBA versus *Col1a2* ^*+/G610C*^ 4‐PBA. Bioinformatic analyses were carried out by Cluego software (Cytoskape release 3.8.2) to cluster enriched annotation groups of biological processes, pathways, and networks within the set of differentially expressed or exclusively expressed proteins in *Col1a1* ^*+/+*^ versus *Col1a2* ^*+/+*^, *Col1a1* ^*+/+*^ 4‐PBA versus *Col1a2* ^*+/+*^ 4‐PBA, *Col1a1* ^*+/G349C*^ versus *Col1a2* ^*+/G610C*^, *Col1a1* ^*+/G349C*^ 4‐PBA versus *Col1a2* ^*+/G610C*^ 4‐PBA. Functional grouping was based on *p* ≤ 0.05. GO term fusion allowed and at least 3 genes count. Proteins were considered differentially expressed in the comparison if they showed significant *t*‐test difference (FDR ≤ 0.05) or were expressed exclusively in one condition.
**Figure S3:** Panther pathways analysis of the proteins differentially or exclusively expressed in *Col1a1* ^*+/+*^ versus *Col1a2* ^*+/+*^, *Col1a1* ^*+/+*^ 4‐PBA versus *Col1a2* ^*+/+*^ 4‐PBA, *Col1a1* ^*+/G349C*^ versus *Col1a2* ^*+/G610C*^, *Col1a1* ^*+/G349C*^ 4‐PBA versus *Col1a2* ^*+/G610C*^ 4‐PBA. Bioinformatic analyses were carried out by Panther software (release 16.0) to cluster enriched Panther pathway within the set of differentially expressed or exclusively expressed proteins in *Col1a1* ^*+/+*^ versus *Col1a2* ^*+/+*^, *Col1a1* ^*+/+*^ 4‐PBA versus *Col1a2* ^*+/+*^ 4‐PBA, *Col1a1* ^*+/G349C*^ versus *Col1a2* ^*+/G610C*^, *Col1a1* ^*+/G349C*^ 4‐PBA versus *Col1a2* ^*+/G610C*^ 4‐PBA. Proteins were considered differentially expressed in the comparison if they showed significant *t*‐test difference (FDR ≤ 0.05) or were expressed exclusively in one condition. If any Panther pathways enrichment was found the data were processed by Panther Reactome to find Reactome GO and pathways enrichment. Functional grouping was based on Fischer's exact test (*p* ≤ 0.05).

**Figure S4:** High‐resolution file allowing detailed zooming of the bioinformatic analyses of the qPCR‐based transcriptome in *Col1a1* ^*+/G349C*^ osteoblasts, as shown in Figure 3B (upper panel). The analyses revealed the presence of hub genes, including *P53*. The high‐resolution file enables detailed zooming, allowing the names of all proteins to be clearly visualized.

**Figure S5:** High‐resolution file allowing detailed zooming of the bioinformatic analyses of the qPCR‐based transcriptome in *Col1a2* ^*+/G610C*^ osteoblasts, as shown in Figure 3B (lower panel). The analyses revealed the presence of hub genes, including *P53*. The high‐resolution file enables detailed zooming, allowing the names of all proteins to be clearly visualized.

**Table S1:** List of the proteins exclusively expressed in Col1a1^+/+^.
**Table S2:** List of the proteins exclusively expressed in Col1a1+/+ 4‐PBA.
**Table S3:** List of the proteins exclusively expressed in Col1a1+/G349C.
**Table S4:** List of the proteins exclusively expressed in Col1a1+/G349C 4‐PBA.
**Table S5:** List of the proteins exclusively expressed in Col1a2+/+.
**Table S6:** List of the proteins exclusively expressed in Col1a2+/+ 4‐PBA.
**Table S7:** List of the proteins exclusively expressed in Col1a2+/G610C.
**Table S8:** List of the proteins exclusively expressed in Col1a2+/G610C 4‐PBA.

**Table S9:** List of the proteins differentially expressed in Col1a1+/G349C versus Col1a1+/+. Proteins were considered differentially expressed in the comparison if they showed significant *t*‐test difference (FDR ≤ 0.05) or are expressed exclusively in one condition.
**Table S10:** List of the proteins differentially expressed in Col1a1+/G349C 4‐PBA versus Col1a1+/+ 4‐PBA Proteins were considered differentially expressed in the comparison if they showed significant *t*‐test difference (FDR ≤ 0.05) or are expressed exclusively in one condition.
**Table S11:** List of the proteins differentially expressed in Col1a1+/G349C 4‐PBA versus Col1a1+/+. Proteins were considered differentially expressed in the comparison if they showed significant *t*‐test difference (FDR ≤ 0.05) or are expressed exclusively in one condition.
**Table S12:** List of the proteins differentially expressed in Col1a2+/G610C versus Col1a2+/+. Proteins were considered differentially expressed in the comparison if they showed significant *t*‐test difference (FDR ≤ 0.05) or are expressed exclusively in one condition.
**Table S13:** List of the proteins differentially expressed in Col1a2+/G610C 4‐PBA versus Col1a2+/+ 4‐PBA. Proteins were considered differentially expressed in the comparison if they showed significant *t*‐test difference (FDR ≤ 0.05) or are expressed exclusively in one condition.
**Table S14:** List of the proteins differentially expressed in Col1a2+/G610C 4‐PBA versus Col1a2+/+. Proteins were considered differentially expressed in the comparison if they showed significant *t*‐test difference (FDR ≤ 0.05) or are expressed exclusively in one condition.

**Table S15:** List of the proteins differentially expressed in Col1a1+/+ versus Col1a2+/+. Proteins were considered differentially expressed in the comparison if they showed significant *t*‐test difference (FDR ≤ 0.05) or are expressed exclusively in one condition.
**Table S16:** List of the proteins differentially expressed in Col1a1+/+ 4‐PBA versus Col1a2+/+ 4‐PBA Proteins were considered differentially expressed in the comparison if they showed significant *t*‐test difference (FDR ≤ 0.05) or are expressed exclusively in one condition.
**Table S17:** List of the proteins differentially expressed in Col1a1+/G349C versus Col1a2+/G610C. Proteins were considered differentially expressed in the comparison if they showed significant *t*‐test difference (FDR ≤ 0.05) or are expressed exclusively in one condition.
**Table S18:** List of the proteins differentially expressed in Col1a1+/G349C 4‐PBA versus Col1a2+/G610C 4‐PBA. Proteins were considered differentially expressed in the comparison if they showed significant *t*‐test difference (FDR ≤ 0.05) or are expressed exclusively in one condition.

**Table S19:** Bioinformatic analysis by Cluego of the proteins differentially or exclusively expressed in *Col1a1* ^*+/+*^ vs *Col1a2* ^*+/+*^, *Col1a1* ^*+/+*^ 4‐PBA vs *Col1a2* ^*+/+*^ 4‐PBA, *Col1a1* ^*+/G349C*^ versus *Col1a2* ^*+/G610C*^, *Col1a1* ^*+/G349C*^ 4‐PBA versus *Col1a2* ^*+/G610C*^ 4‐PBA. Bioinformatic analyses were carried out by Cluego software (Cytoskape release 3.8.2) within the set of differentially expressed or exclusively expressed proteins in the comparisons listed above. Functional grouping was based on *p* ≤ 0.05, GO terms fusion allowed and at least 3 counts.

**Table S20:** Panther analysis of the proteins differentially expressed in *Col1a1* ^*+/+*^ versus *Col1a2* ^*+/+*^, *Col1a1* ^*+/+*^ 4‐PBA versus *Col1a2* ^*+/+*^ 4‐PBA, *Col1a1* ^*+/G349C*^ versus *Col1a2* ^*+/G610C*^, *Col1a1* ^*+/G349C*^ 4‐PBA versus *Col1a2* ^*+/G610C*^ 4‐PBA. Proteins differentially expressed in the comparisons were analysed by Panther (release 16.0) for pathways enrichment. If any Panther pathways enrichment was found the data were processed by Panther Reactome to find Reactome GO and pathways enrichment. The column “Counts” indicates the number of genes present in each category. Functional grouping was based on Fischer's exact test (*p* ≤ 0.05).

## Data Availability

The mass spectrometry proteomics data have been deposited to the ProteomeXchange Consortium via PRIDE (PXD059591).
