# Supplementary material for: OMICS Profiling Identifies Signatures of Senescence in Osteogenesis Imperfecta Osteoblasts Counteracted by 4‐PBA
Source: J Cell Mol Med. 2026 Apr 6;30(7):e71120. doi: 10.1111/jcmm.71120 (PMC13052117; doi:10.1111/jcmm.71120)
Supplement: Supplementary file 1 — Figure S1: (A, B) Scatter plots of the correlations between the three biological replicates in all the comparisons. Figure S2: Bioinformatic analysis by Cluego of the proteins differentially or exclusively expressed in Col1a1 +/+ versus Col1a2 +/+ , Col1a1 +/+ 4‐PBA versus Col1a2 +/+ 4‐PBA, Col1a1 +/G349C versus Col1a2 +/G610C , Col1a1 +/G349C 4‐PBA versus Col1a2 +/G610C 4‐PBA. Bioinformatic analyses were carried out by Cluego software (Cytoskape release 3.8.2) to cluster enriched annotation groups of biological processes, pathways, and networks within the set of differentially expressed or exclusively expressed proteins in Col1a1 +/+ versus Col1a2 +/+ , Col1a1 +/+ 4‐PBA versus Col1a2 +/+ 4‐PBA, Col1a1 +/G349C versus Col1a2 +/G610C , Col1a1 +/G349C 4‐PBA versus Col1a2 +/G610C 4‐PBA. Functional grouping was based on p ≤ 0.05. GO term fusion allowed and at least 3 genes count. Proteins were considered differentially expressed in the comparison if they showed significant t‐test difference (FDR ≤ 0.05) or were expressed exclusively in one condition. Figure S3: Panther pathways analysis of the proteins differentially or exclusively expressed in Col1a1 +/+ versus Col1a2 +/+ , Col1a1 +/+ 4‐PBA versus Col1a2 +/+ 4‐PBA, Col1a1 +/G349C versus Col1a2 +/G610C , Col1a1 +/G349C 4‐PBA versus Col1a2 +/G610C 4‐PBA. Bioinformatic analyses were carried out by Panther software (release 16.0) to cluster enriched Panther pathway within the set of differentially expressed or exclusively expressed proteins in Col1a1 +/+ versus Col1a2 +/+ , Col1a1 +/+ 4‐PBA versus Col1a2 +/+ 4‐PBA, Col1a1 +/G349C versus Col1a2 +/G610C , Col1a1 +/G349C 4‐PBA versus Col1a2 +/G610C 4‐PBA. Proteins were considered differentially expressed in the comparison if they showed significant t‐test difference (FDR ≤ 0.05) or were expressed exclusively in one condition. If any Panther pathways enrichment was found the data were processed by Panther Reactome to find Reactome GO and pathways enrichment. Functional grouping [file JCMM-30-e71120-s001.docx]

**Supplementary Material**

**Supplementary Figures**

**Figure S1A and B. Scatter plots of the correlations between the three biological replicates in all the comparisons.**

**
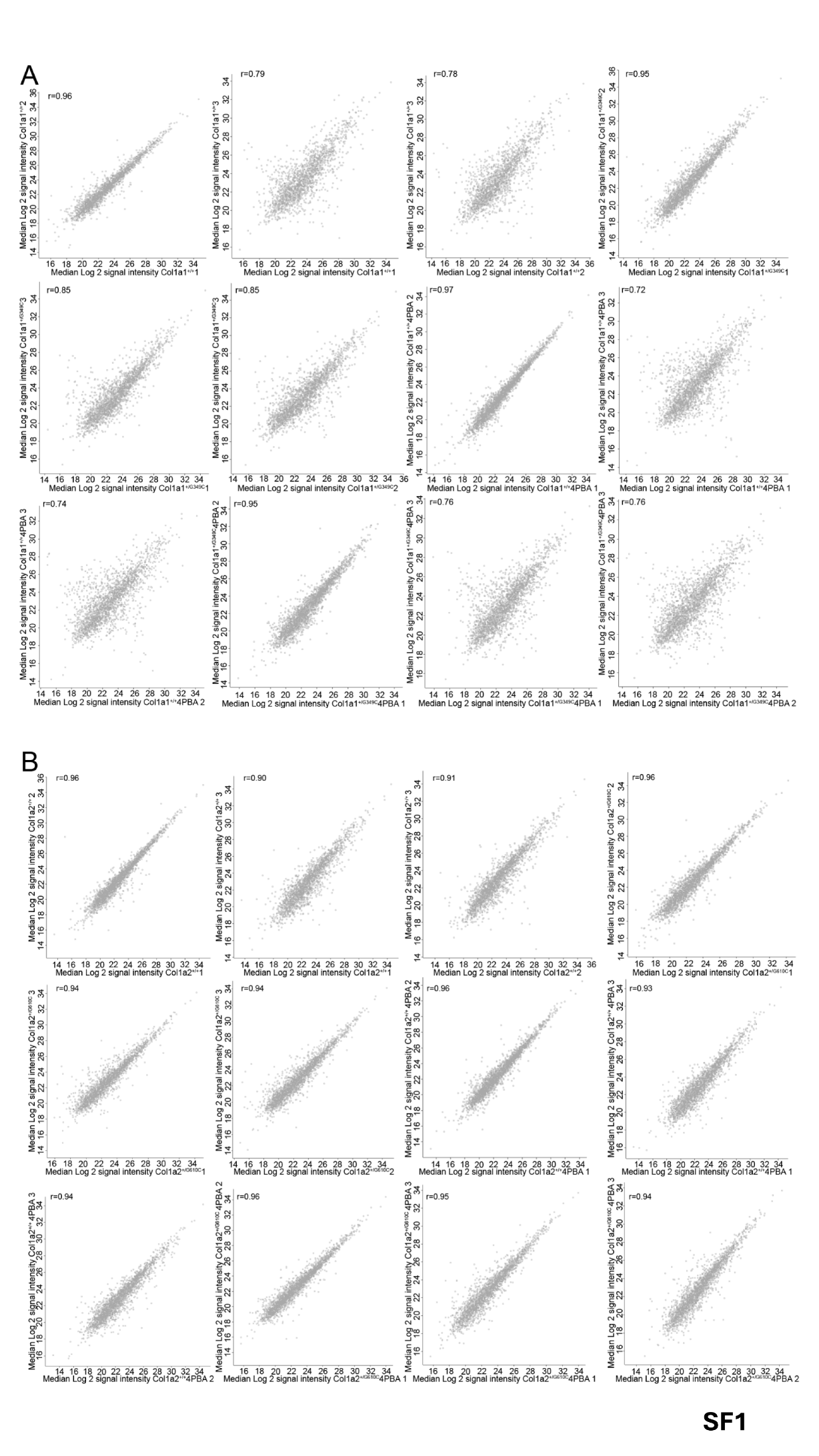
**

**Figure S2**. **Bioinformatic analysis by Cluego of the proteins differentially or exclusively expressed in** ***Col1a1^+/+^* vs *Col1a2^+/+^*, *Col1a1^+/+^* 4-PBA vs *Col1a2^+/+^* 4-PBA, *Col1a1^+/G349C^* vs *Col1a2^+/G610C^*, *Col1a1^+/G349C^* 4-PBA vs *Col1a2^+/G610C^* 4-PBA**. Bioinformatic analyses were carried out by Cluego software (Cytoskape release 3.8.2) to cluster enriched annotation groups of biological processes, pathways, and networks within the set of differentially expressed or exclusively expressed proteins in *Col1a1^+/+^* vs *Col1a2^+/+^*, *Col1a1^+/+^* 4-PBA vs *Col1a2^+/+^* 4-PBA, *Col1a1^+/G349C^* vs *Col1a2^+/G610C^*, *Col1a1^+/G349C^* 4-PBA vs *Col1a2^+/G610C^* 4-PBA. Functional grouping was based on p ≤ 0.05. GO term fusion allowed and at least 3 genes count. Proteins were considered differentially expressed in the comparison if they showed significant t-test difference (FDR ≤0.05) or were expressed exclusively in one condition.

**
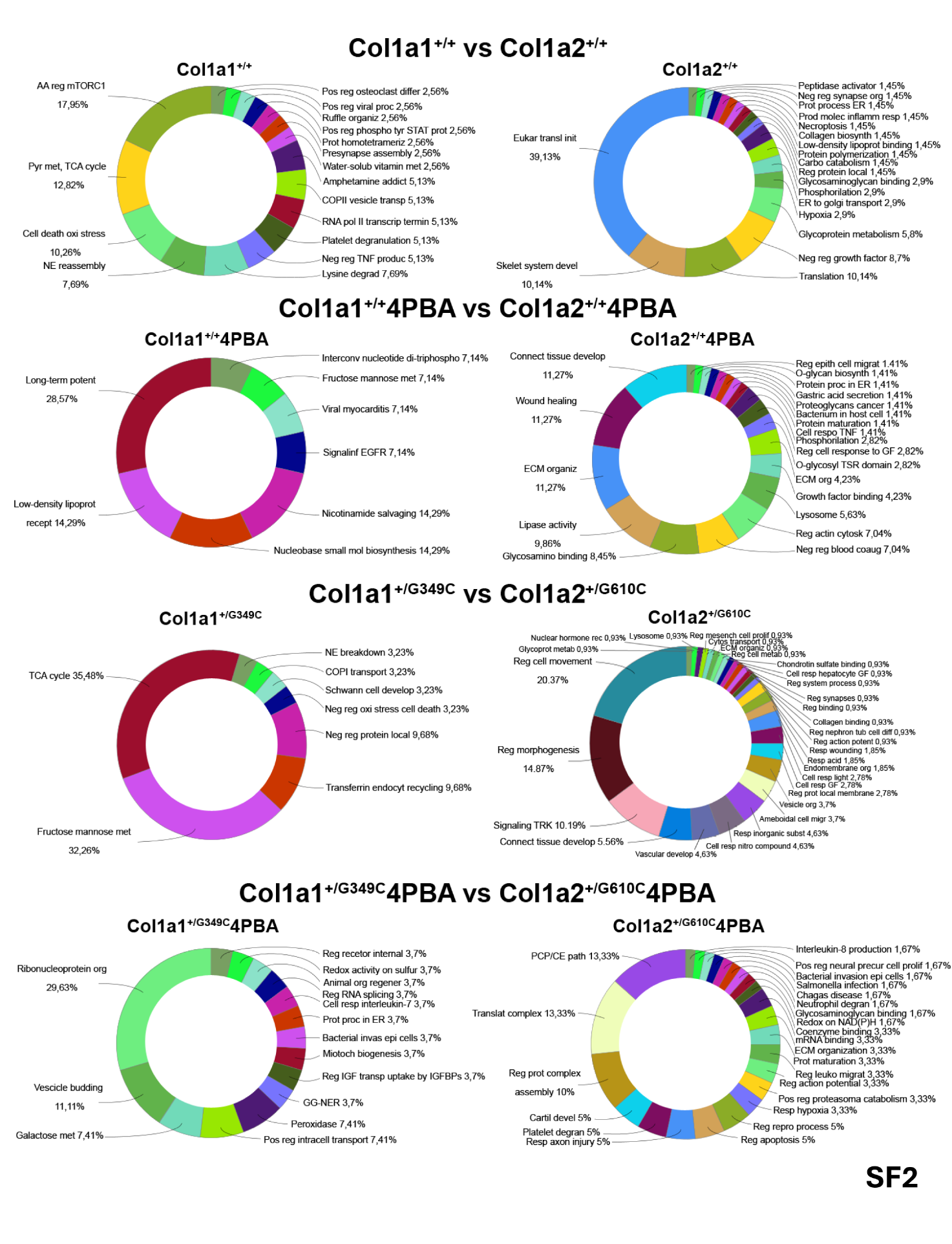
**

**Figure S3. Panther pathways analysis of the proteins differentially or exclusively expressed in *Col1a1^+/+^* vs *Col1a2^+/+^,* *Col1a1^+/+^* 4-PBA vs *Col1a2^+/+^* 4-PBA, *Col1a1^+/G349C^* vs *Col1a2^+/G610C^*, *Col1a1^+/G349C^* 4-PBA vs *Col1a2^+/G610C^* 4-PBA**. Bioinformatic analyses were carried out by Panther software (release 16.0) to cluster enriched Panther pathway within the set of differentially expressed or exclusively expressed proteins in *Col1a1^+/+^* vs *Col1a2^+/+^,* *Col1a1^+/+^* 4-PBA vs *Col1a2^+/+^* 4-PBA, *Col1a1^+/G349C^* vs *Col1a2^+/G610C^*, *Col1a1^+/G349C^* 4-PBA vs *Col1a2^+/G610C^* 4-PBA. Proteins were considered differentially expressed in the comparison if they showed significant t-test difference (FDR ≤0.05) or were expressed exclusively in one condition. If any Panther pathways enrichment was found the data were processed by Panther Reactome to find Reactome GO and pathways enrichment. Functional grouping was based on Fischer’s exact test (p ≤ 0.05).

**
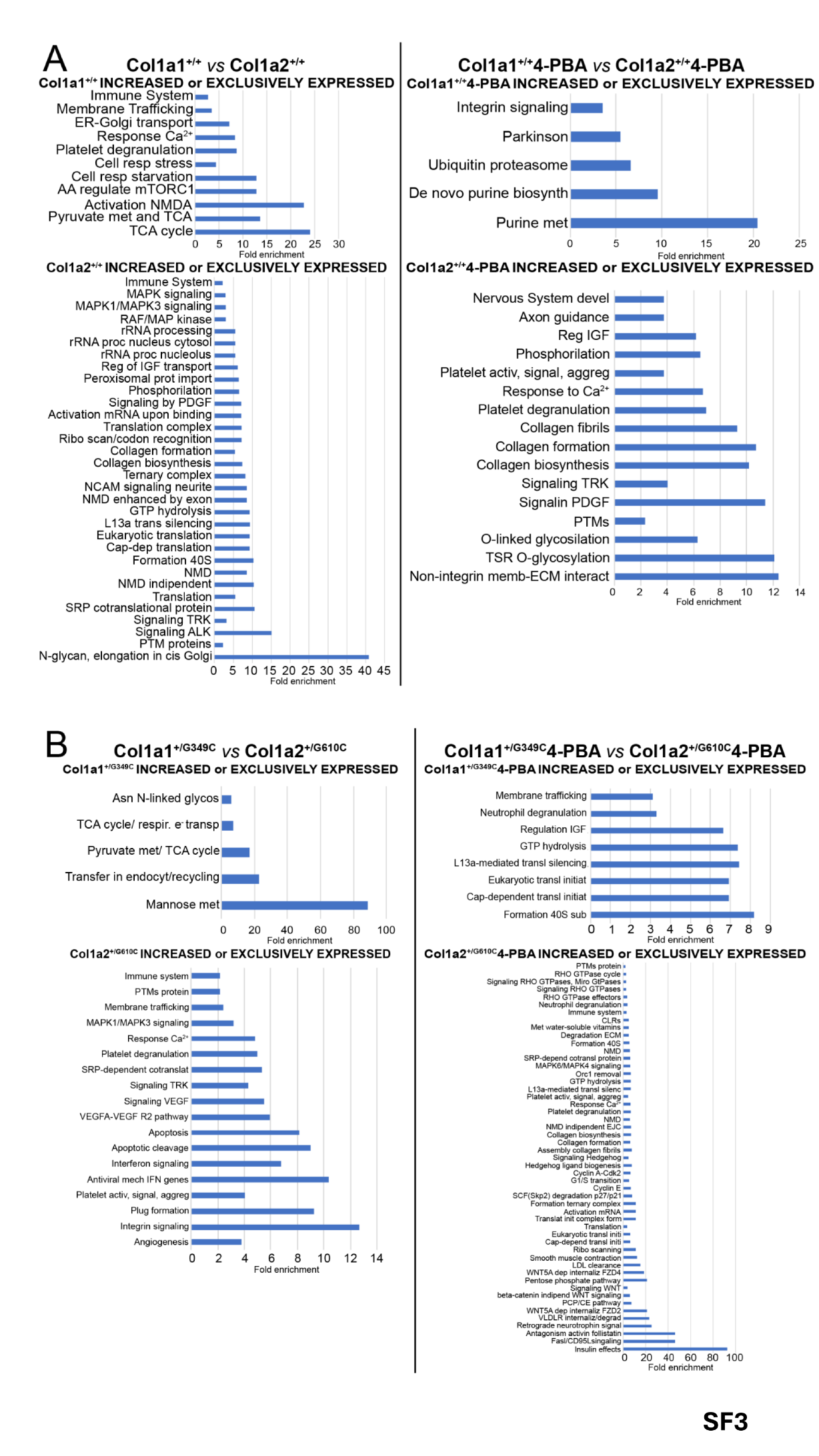
**

**Figure S4. High-resolution file allowing detailed zooming of the bioinformatic analyses of the qPCR-based transcriptome in *Col1a1^+/G349C^* osteoblasts, as shown in Figure 3B (upper panel).** The analyses revealed the presence of hub genes, including *P53*. The high-resolution file enables detailed zooming, allowing the names of all proteins to be clearly visualized.

**Figure S5. High-resolution file allowing detailed zooming of the bioinformatic analyses of the qPCR-based transcriptome in *Col1a2^+/G610C^* osteoblasts, as shown in Figure 3B (lower panel).** The analyses revealed the presence of hub genes, including *P53*. The high-resolution file enables detailed zooming, allowing the names of all proteins to be clearly visualized.

**Supplementary Tables**

**Table S1-S8**

**S1: List of the proteins exclusively expressed in *Col1a1^+/+^*.** Proteins commonly expressed in the data sets *Col1a1^+/+^*, *Col1a1^+/+^* 4-PBA, *Col1a^1+/G349C^*, *Col1a1^+/G349C^* 4-PBA, *Col1a2^+/+^*, *Col1a2^+/+^* 4-PBA, Col1a2^+/G610C^, *Col1a2^+/G610C^* 4-PBA and proteins exclusively present in each one of them were identified by one-way ANOVA analysis followed by post-hoc tests with the Bonferroni’s correction (p value≤ 0.05) (Fig. 1C). The Table reports the proteins ANOVA significant exclusively expressed in *Col1a1^+/+^.*

**S2: List of the proteins exclusively expressed in *Col1a1^+/+^* 4-PBA.** Proteins commonly expressed in the data sets *Col1a1^+/+^*, *Col1a1^+/+^* 4-PBA, *Col1a1^+/G349C^*, *Col1a1^+/G349C^* 4-PBA, *Col1a2^+/+^*, *Col1a2^+/+^* 4-PBA, *Col1a2^+/G610C^*, *Col1a2^+/G610C^* 4-PBA and proteins exclusively present in each one of them were identified by one-way ANOVA analysis followed by post-hoc tests with the Bonferroni’s correction (p value≤ 0.05) (Fig. 1C). The Table reports the proteins ANOVA significant exclusively expressed in *Col1a1^+/+^* 4-PBA.

**S3: List of the proteins exclusively expressed in *Col1a1^+/G349C^*.** Proteins commonly expressed in the data sets *Col1a1^+/+^*, *Col1a1^+/+^* 4-PBA, *Col1a1^+/G349C^*, *Col1a1^+/G349C^* 4-PBA, *Col1a2^+/+^*, *Col1a2^+/+^* 4-PBA, *Col1a2^+/G610C^*, *Col1a2^+/G610C^* 4-PBA and proteins exclusively present in each one of them were identified by one-way ANOVA analysis followed by post-hoc tests with the Bonferroni’s correction (p value≤ 0.05) (Fig. 1C). The Table reports the proteins ANOVA significant exclusively expressed in *Col1a1^+/G349C^*.

**S4: List of the proteins exclusively expressed in *Col1a1^+/G349C^* 4-PBA.** Proteins commonly expressed in the data sets *Col1a1^+/+^*, *Col1a1^+/+^* 4-PBA, *Col1a1^+/G349C^*, *Col1a1^+/G349C^* 4-PBA, *Col1a2^+/+^*, *Col1a2^+/+^* 4-PBA, *Col1a2^+/G610C^*, *Col1a2^+/G610C^* 4-PBA and proteins exclusively present in each one of them were identified by one-way ANOVA analysis followed by post-hoc tests with the Bonferroni’s correction (p value≤ 0.05) (Fig. 1C). The Table reports the proteins ANOVA significant exclusively expressed in *Col1a1^+/G349C^* 4-PBA.

**S5: List of the proteins exclusively expressed in Col1a^2+/+^.** Proteins commonly expressed in the data sets *Col1a1^+/+^*, *Col1a1^+/+^* 4-PBA, *Col1a^1+/G349C^*, *Col1a1^+/G349C^* 4-PBA, *Col1a2^+/+^*, *Col1a2^+/+^* 4-PBA, *Col1a2^+/G610C^*, *Col1a2^+/G610C^* 4-PBA and proteins exclusively present in each one of them were identified by one-way ANOVA analysis followed by post-hoc tests with the Bonferroni’s correction (p value≤ 0.05) (Fig. 1C). The Table reports the proteins ANOVA significant exclusively expressed in *Col1a2^+/+^*.

**S6: List of the proteins exclusively expressed in *Col1a2^+/+^* 4-PBA.** Proteins commonly expressed in the data sets *Col1a1^+/+^*, *Col1a1^+/+^* 4-PBA, *Col1a1^+/G349C^*, Col1a1^+/^*^G349C^* 4-PBA, *Col1a2^+/+^*, *Col1a2^+/+^* 4-PBA, *Col1a2^+/G610C^*, *Col1a2^+/G610C^* 4-PBA and proteins exclusively present in each one of them were identified by one-way ANOVA analysis followed by post-hoc tests with the Bonferroni’s correction (p value≤ 0.05) (Fig. 1C). The Table reports the proteins ANOVA significant exclusively expressed in *Col1a2^+/+^* 4-PBA.

**S7: List of the proteins exclusively expressed in *Col1a2^+/G610C^*.** Proteins commonly expressed in the data sets *Col1a1^+/+^, Col1a1^+/+^* 4-PBA, *Col1a1^+/G349C^*, *Col1a1^+/G349C^* 4-PBA, *Col1a2^+/+^, Col1a2^+/+^* 4-PBA, *Col1a2^+/G610C^, Col1a2^+/G610C^* 4-PBA and proteins exclusively present in each one of them were identified by one-way ANOVA analysis followed by post-hoc tests with the Bonferroni’s correction (p value≤ 0.05) (Fig. 1C). The Table reports the proteins ANOVA significant exclusively expressed in *Col1a2^+/G610C^*.

**S8: List of the proteins exclusively expressed in *Col1a2^+/G610C^* 4-PBA.** Proteins commonly expressed in the data sets *Col1a1^+/+^, Col1a1^+/+^* 4-PBA, *Col1a1^+/G349C^*, *Col1a1^+/G349C^* 4-PBA, *Col1a2^+/+^*, *Col1a2^+/+^* 4-PBA, *Col1a2^+/G610C^*, *Col1a2^+/G610C^* 4-PBA and proteins exclusively present in each one of them were identified by one-way ANOVA analysis followed by post-hoc tests with the Bonferroni’s correction (p value≤ 0.05) (Fig. 1C). The Table reports the proteins ANOVA significant exclusively expressed in *Col1a2^+/G610C^* 4-PBA.

**Table S9-S14**

**S9: List of the proteins differentially expressed in *Col1a1^+/G349C^* vs *Col1a1^+/+^*.** Proteins were considered differentially expressed in the comparison if they showed significant t-test difference (FDR ≤0.05) or are expressed exclusively in one condition.

**S10 List of the proteins differentially expressed in *Col1a1^+/G349C^* 4-PBA vs *Col1a1^+/+^* 4-PBA.** Proteins were considered differentially expressed in the comparison if they showed significant t-test difference (FDR ≤0.05) or are expressed exclusively in one condition.

**S11 List of the proteins differentially expressed in *Col1a1*^+/G349C^ 4-PBA vs *Col1a1*^+/+^.** Proteins were considered differentially expressed in the comparison if they showed significant t-test difference (FDR ≤0.05) or are expressed exclusively in one condition.

**S12 List of the proteins differentially expressed in *Col1a2^+^*^/G610C^ vs *Col1a2^+^*^/+^.** Proteins were considered differentially expressed in the comparison if they showed significant t-test difference (FDR ≤0.05) or are expressed exclusively in one condition.

**S13 List of the proteins differentially expressed in *Col1a2^+^*^/G610C^ 4-PBA vs *Col1a2^+^*^/+^ 4-PBA.** Proteins were considered differentially expressed in the comparison if they showed significant t-test difference (FDR ≤0.05) or are expressed exclusively in one condition.

**S14 List of the proteins differentially expressed in *Col1a2^+^*^/G610C^ 4-PBA vs *Col1a2^+^*^/+^.** Proteins were considered differentially expressed in the comparison if they showed significant t-test difference (FDR ≤0.05) or are expressed exclusively in one condition.

**Table S15-S18**

**S15: List of the proteins differentially expressed in *Col1a1^+/+^* vs *Col1a2^+/+^*.** Proteins were considered differentially expressed in the comparison if they showed significant t-test difference (FDR ≤0.05) or are expressed exclusively in one condition.

**S16: List of the proteins differentially expressed in *Col1a1^+/+^* 4-PBA vs *Col1a2^+/+^* 4-PBA** Proteins were considered differentially expressed in the comparison if they showed significant t-test difference (FDR ≤0.05) or are expressed exclusively in one condition.

**S17: List of the proteins differentially expressed in *Col1a1^+/G349C^* vs *Col1a2^+/G610C^*.** Proteins were considered differentially expressed in the comparison if they showed significant t-test difference (FDR ≤0.05) or are expressed exclusively in one condition.

**S18: List of the proteins differentially expressed in *Col1a1^+/G349C^* 4-PBA vs *Col1a2^+/G610C^* 4-PBA**. Proteins were considered differentially expressed in the comparison if they showed significant t-test difference (FDR ≤0.05) or are expressed exclusively in one condition.

**Table S19: Bioinformatic analysis by Cluego of the proteins differentially or exclusively expressed in *Col1a1^+/+^* vs *Col1a2^+/+^*, *Col1a1^+/+^* 4-PBA vs *Col1a2^+/+^* 4-PBA, *Col1a1^+/G349C^* vs *Col1a2^+/G610C^*,** ***Col1a1^+/G349C^* 4-PBA vs *Col1a2^+/G610C^* 4-PBA.** Bioinformatic analyses were carried out by Cluego software (Cytoskape release 3.8.2) within the set of differentially expressed or exclusively expressed proteins in the comparisons listed above. Functional grouping was based on P ≤ 0.05, GO terms fusion allowed and at least 3 counts.

**Table S20: Panther analysis of the proteins differentially expressed in *Col1a1^+/+^* vs *Col1a2^+/+^*, *Col1a1^+/+^* 4-PBA vs *Col1a2^+/+^* 4-PBA, *Col1a1^+/G349C^* vs *Col1a2^+/G610C^*,** ***Col1a1^+/G349C^* 4-PBA vs *Col1a2^+/G610C^* 4-PBA.** Proteins differentially expressed in the comparisons were analysed by Panther (release 16.0) for pathways enrichment. If any Panther pathways enrichment was found the data were processed by Panther Reactome to find Reactome GO and pathways enrichment. The column “Counts” indicates the number of genes present in each category. Functional grouping was based on Fischer’s exact test (p ≤ 0.05).
