# Supplementary figures and images for: OMICS Profiling Identifies Signatures of Senescence in Osteogenesis Imperfecta Osteoblasts Counteracted by 4‐PBA
Source: J Cell Mol Med. 2026 Apr 6;30(7):e71120. doi: 10.1111/jcmm.71120 (PMC13052117; doi:10.1111/jcmm.71120)

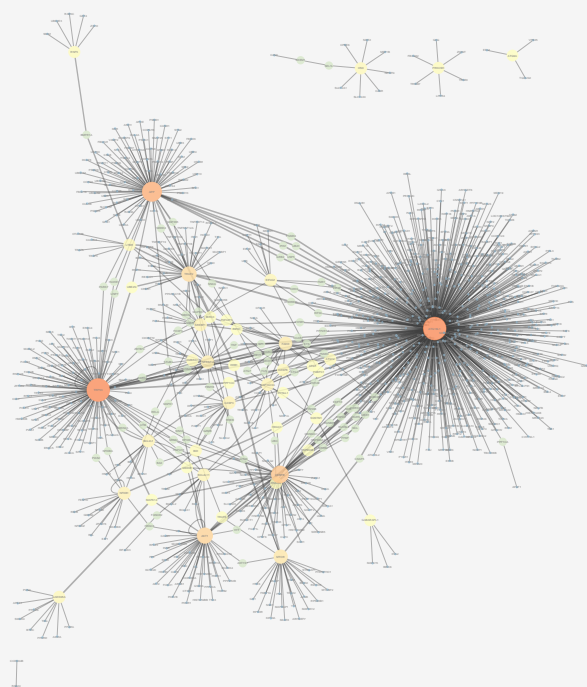

Supplement: Supplementary file 2 — Figure S4: High‐resolution file allowing detailed zooming of the bioinformatic analyses of the qPCR‐based transcriptome in Col1a1 +/G349C osteoblasts, as shown in Figure 3B (upper panel). The analyses revealed the presence of hub genes, including P53. The high‐resolution file enables detailed zooming, allowing the names of all proteins to be clearly visualized. [file JCMM-30-e71120-s008.pdf]

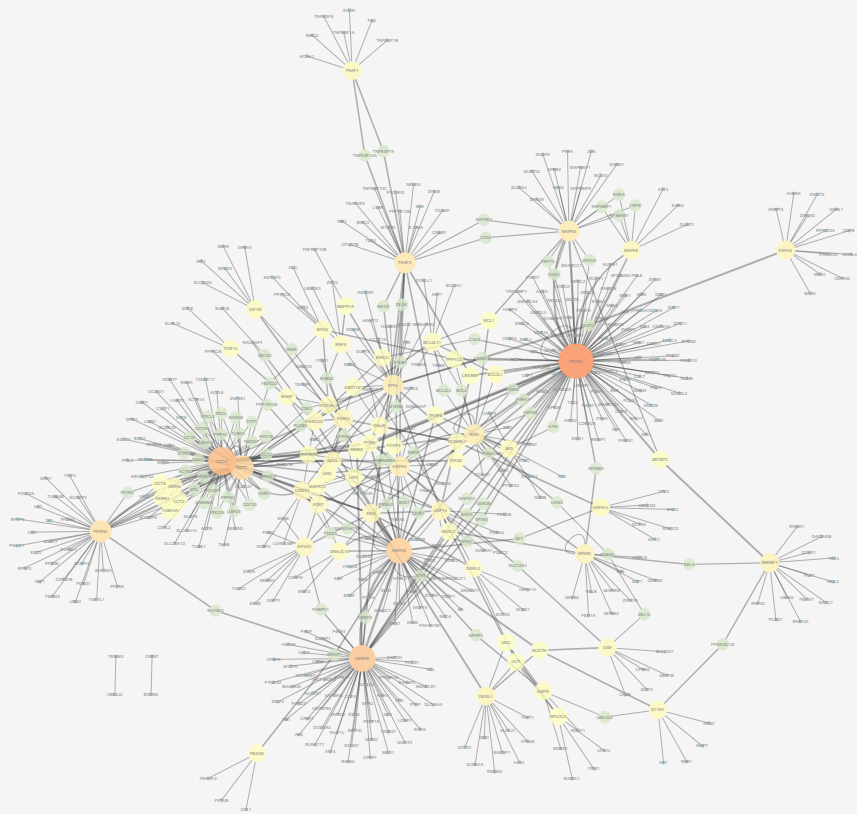

Supplement: Supplementary file 3 — Figure S5: High‐resolution file allowing detailed zooming of the bioinformatic analyses of the qPCR‐based transcriptome in Col1a2 +/G610C osteoblasts, as shown in Figure 3B (lower panel). The analyses revealed the presence of hub genes, including P53. The high‐resolution file enables detailed zooming, allowing the names of all proteins to be clearly visualized. [file JCMM-30-e71120-s003.pdf]
